# Assessing proxy and AI models performance in waterflooding optimization

**DOI:** 10.1038/s41598-025-19256-4

**Published:** 2025-10-10

**Authors:** Yaser Abdollahfard, Jalal Fahimpour, Mohammad Ahmadi

**Affiliations:** 1 Petroleum Engineering Department, Amirkabir University of Technology, Tehran, Iran; 2 Institute of GeoEnergy Engineering, Heriot-Watt University, Edinburgh, Scotland

**Keywords:** Water flooding optimization, Proxy models, Net present value, Deep learning, And particle swarm optimization, Geology, Fluid dynamics

## Abstract

Water flooding remains one of the most widely used improved oil recovery (IOR) methods, even after decades of hydrocarbon production and the application of various IOR techniques. However, real reservoir simulations are computationally expensive and time-consuming due to the complexity of reservoir models. To address this, researchers increasingly rely on artificial intelligence and neural network-based proxy models as faster alternatives. While proxy models reduce computational effort, their ability to predict reservoir behavior remains a challenge reliably. This study evaluates the reliability of proxy models in optimization tasks. The Punq-S3 benchmark reservoir model was used to optimize well control parameters and maximize net present value (NPV) through two approaches: one based on proxy models and the other on full-physics reservoir simulation. Four deep learning algorithms (ANN, LSTM, GRU, and EL) were combined with two design of experiment techniques (Taguchi and Latin hypercube sampling) to generate eight proxy models. The particle swarm optimization and Bayesian optimization algorithms were selected to optimize the injection and production strategy for the two approaches. after optimization and comparison of the results, it was observed that despite the constructed proxy models exhibited acceptable predictive performance in terms of statistical metrics (e.g., RMSE, R²), the optimization results they yielded significantly deviated from those based on the full-physics reservoir model. This reveals a critical trade-off between computational efficiency and decision-making reliability. While proxy models offer a cost-effective alternative for rapid predictions, caution is needed when applying them to optimization tasks, where minor prediction errors can lead to substantially different outcomes. This study highlights the importance of evaluating proxy models not only based on accuracy metrics but also on their ability to capture optimization-relevant dynamics.

## Introduction

After a specific period of oil production by natural lift mechanism, oil reservoirs gradually encounter the reservoir’s average pressure reduction and require the operations of the secondary recovery. Water flooding is one of the inexpensive secondary recovery operations to reservoir pressure maintenance^1^.

Water flooding is a frequently utilized method for enhanced and improved oil recovery (EOR and IOR), implemented through various techniques, such as seawater flooding, low salinity water flooding, and smart water flooding. In the water flooding process, water is injected into a reservoir, which facilitates the mobilization of residual oil towards production wells while maintaining reservoir pressure. If low salinity or smart water is introduced, it can improve the wettability of reservoir rock and oil/water mobility during this process and enhance oil recovery. The importance of using water flooding can be summarized as follows:

1. Reservoir Pressure Maintenance: Maintaining reservoir pressure is crucial for sustaining oil production rates. Water injection helps to counteract the natural decline in pressure as the oil is produced, ensuring that the reservoir remains sufficiently pressurized to facilitate continued flow^2^.
2. Improved Sweep Efficiency: Effective water flooding can enhance sweep efficiency, which refers to the ability of the injected water to displace oil within the reservoir. Optimizing injection strategies can lead to a more uniform displacement front, reducing bypassed oil and improving overall recovery^2^.
3. Cost Efficiency: Optimizing injection-production strategies can lead to significant cost savings by maximizing oil recovery with minimal additional investment. Efficient water flooding can reduce the need for more expensive EOR methods later in the field’s life cycle^2^.
4. Environmental Considerations: Effective management of water flooding operations can help minimize environmental impacts associated with excessive water production and disposal, as well as reduce the carbon footprint of oil extraction processes^2^.

Key parameters affecting water flooding performance are as follows:

1. Injection Rate: The rate at which water is injected into the reservoir is crucial. High injection rates can lead to fracturing and channeling, while low rates may not adequately maintain reservoir pressure. An optimal injection rate balances these effects^3^.
2. Injection Location: The location of injection wells significantly influences the flow path of both water and oil. Strategic placement can enhance sweep efficiency and reduce the risk of water breakthrough^4^.
3. Reservoir Heterogeneity: Reservoirs are rarely homogeneous; variations in porosity, permeability, and fluid saturation can impact how effectively water displaces oil. Understanding the geological characteristics is vital for optimizing water flood patterns^5^.
4. Fluid Properties: The viscosity and density of both the injected water and the reservoir oil play a significant role in displacement efficiency. Lower viscosity fluids tend to yield better mobility ratios, enhancing recovery^6^.
5. Water Quality: The chemical composition of the injected water can affect reservoir rock properties and oil viscosity. Compatibility between injected water and formation water is crucial to prevent issues such as scaling or clay swelling^7^.
6. Operational Strategies: Implementing advanced operational strategies such as variable injection rates, zonal isolation techniques, and real-time monitoring can optimize water flooding performance^8^.

Nowadays, artificial intelligence (AI) is a confident and powerful tool for modeling, predicting, and optimizing. This tool has provided some practical applications in petroleum engineering, e.g., estimation of rock properties (permeability, porosity, etc.)^9,10^ modeling reservoir fluid properties^11,12^ prediction and optimization of shale gas production^13^ reservoir characterization^14,15^ predicting formation damage^16^ digital 3D core reconstruction^17–19^ well test interpretation^20,21^, well log processing^22^ modeling scale, wax and asphaltene deposition^23–25^ the onset of sand production prediction^26^ prediction of deliverability for underground natural gas storage^27^ and reservoir history matching^28,29^.

Identifying and optimizing well-controlled parameters would have a significant impact on the performance of water flooding operations. However, due to the high computational time and cost involved in running real reservoir simulation models, for such optimization processes, proxy models are developed and used. To this end, reservoir engineers usually run a limited number of simulation scenarios, from which they can develop a desired proxy model.

Some examples of such approach are discussed as follow. Alfarizi et al. (2022) used artificial neural networks (ANN) to model Net present value (NPV) at alternative injection and production well control parameters. They optimized it using a Genetic algorithm (GA), and the optimal solution increased NPV by 22.2% compared to the base case^30^. Zhang et al. (2022) proposed double model differential evolution (CSDE) as an optimization algorithm and used a support vector machine algorithm to model NPV. Results demonstrate that the CSDE algorithm can handle constraints more efficiently and achieve higher net present value (NPV) compared with the original evolutionary algorithms and other single-model algorithms^31^. Farahani et al. (2020) utilized two multi-objective optimization algorithms (multi-objective particle swarm optimization (MOPSO) and non-dominated sorting genetic algorithm II (NSGA-II)) to optimize the short- and long-term production strategies in water flooding operations^32^. Ng et al. (2021), with the aid of machine learning method, namely artificial neural networks, established data-driven proxy models that could be utilized to maximize the NPV of a water flooding process by adjusting the well control injection rates over a production period^33^. Ng et al. (2023) demonstrated how long short-term memory (LSTM) algorithm and metaheuristic algorithm, were applied to develop proxies of a 3D reservoir model under water flooding process. They coupled these proxies with particle swarm optimization to conduct production optimization^34^. Liu et al. (2021) developed a water flooding optimization method by running a data-driven inter-well numerical simulation model with flow-path tracking (INSIM-FPT) in three dimensions. They defined producer-centered production efficiency of wells with multiple perforations and propose a rapid water flooding optimization method. The optimized well production rates and injection rates are obtained with the help of the oil cut and the existing injector-centered allocation factors derived from the INSIM model^35^. Ng et al. (2023) surveyed on the employment of ML and the coupled paradigm in proxy modeling of Numerical Reservoir Simulation^36^. Matthew et al. (2023) developed proxy models to solve a multi-objective optimization problem using NSGA-II in two selected reservoir models under CO2-WAG^37^. Jaber et al. (2019) developed a central composite design (CCD) based proxy model for evaluating miscible CO₂-WAG flooding in the Nahr Umr reservoir, validated with 81 simulation runs. The model reliably identified key reservoir and operational parameters influencing incremental oil recovery^38^.

Previous studies have demonstrated the use of machine learning and metaheuristic algorithms to optimize production strategies in waterflooding operations—such as the use of ANN-GA by Alfarizi et al. (2022)^30^ CSDE-SVM by Zhang et al. (2022)^31^ and LSTM-PSO by Ng et al. (2023)^35^—these approaches often focus solely on maximizing economic indicators like NPV without thoroughly assessing the robustness and generalizability of the proxy models under varying operational conditions. The novelty of the present study lies in the integrated evaluation of both the accuracy and robustness of proxy and AI models, specifically under different injection and production strategies.

This study addresses the lack of comprehensive robustness and accuracy evaluations of proxy models in dynamic reservoir optimization, particularly under complex, real-world scenarios. By benchmarking proxy and AI models using the modified PUNQ-S3 reservoir under water flooding, it fills a critical gap in understanding their predictive reliability and adaptability across varying operational conditions. For this purpose, two methods were employed for optimizing the injection and production strategies in a benchmark reservoir model under water flooding process. In one case, several proxy models were constructed through deep learning algorithms, in which the control parameters were optimized using two optimization algorithms. The second case, the real reservoir model was used to run various injection scenarios and find the optimum control parameters by those two optimization algorithms.

## Methodology

**Fig. 1:**
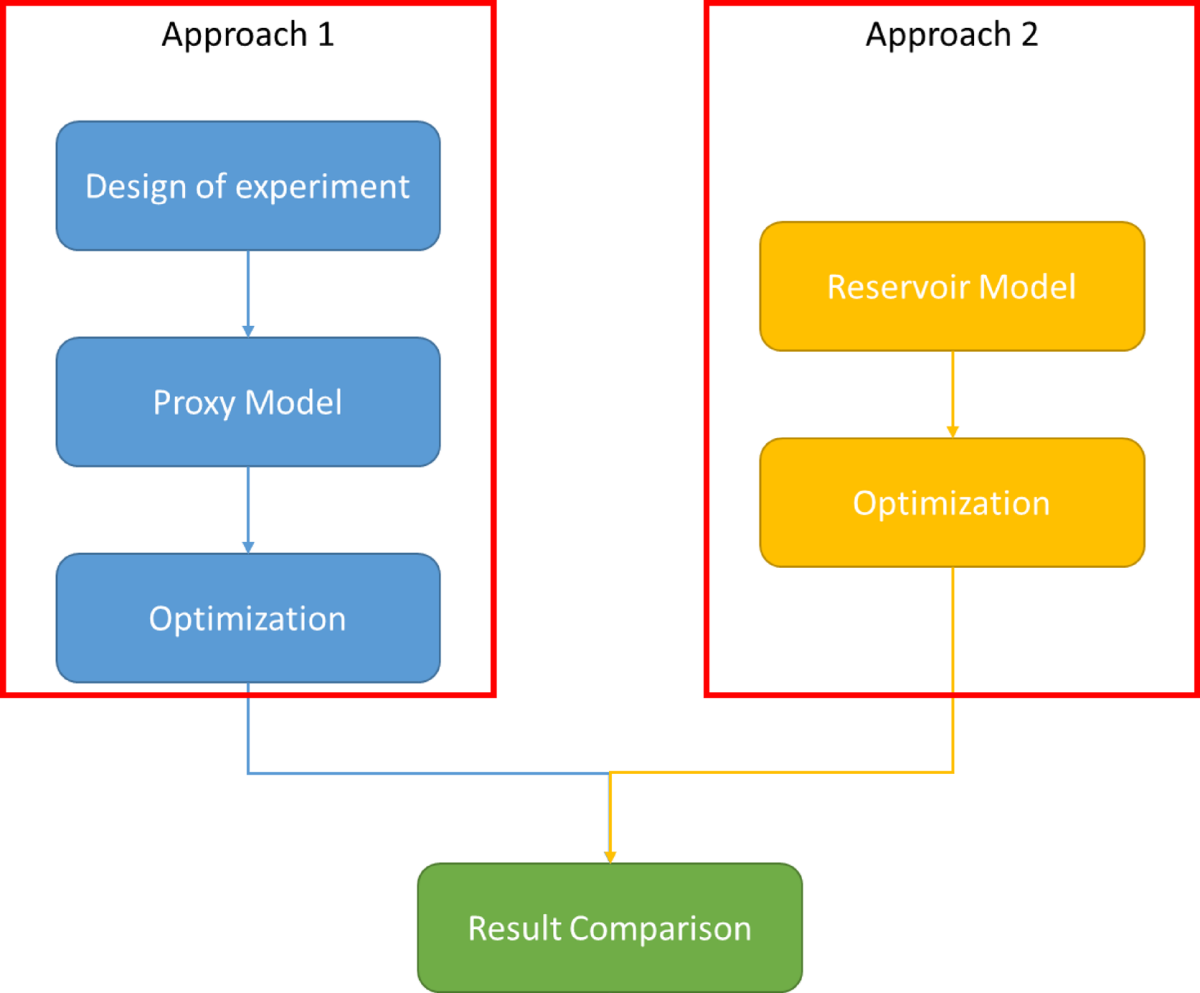
Two approaches for waterflooding optimization.

This study compares two approaches (see Fig. 1) for optimizing injection and production strategies utilizing (1) proxy models and (2) a real reservoir model. The reservoir model employed is the Punq-S3 benchmark model. The input data used in optimization process consist of the oil production rate of producers and the injection pressure of the injectors. The objective of optimization is to enhance the project’s NPV.

**Fig. 2:**
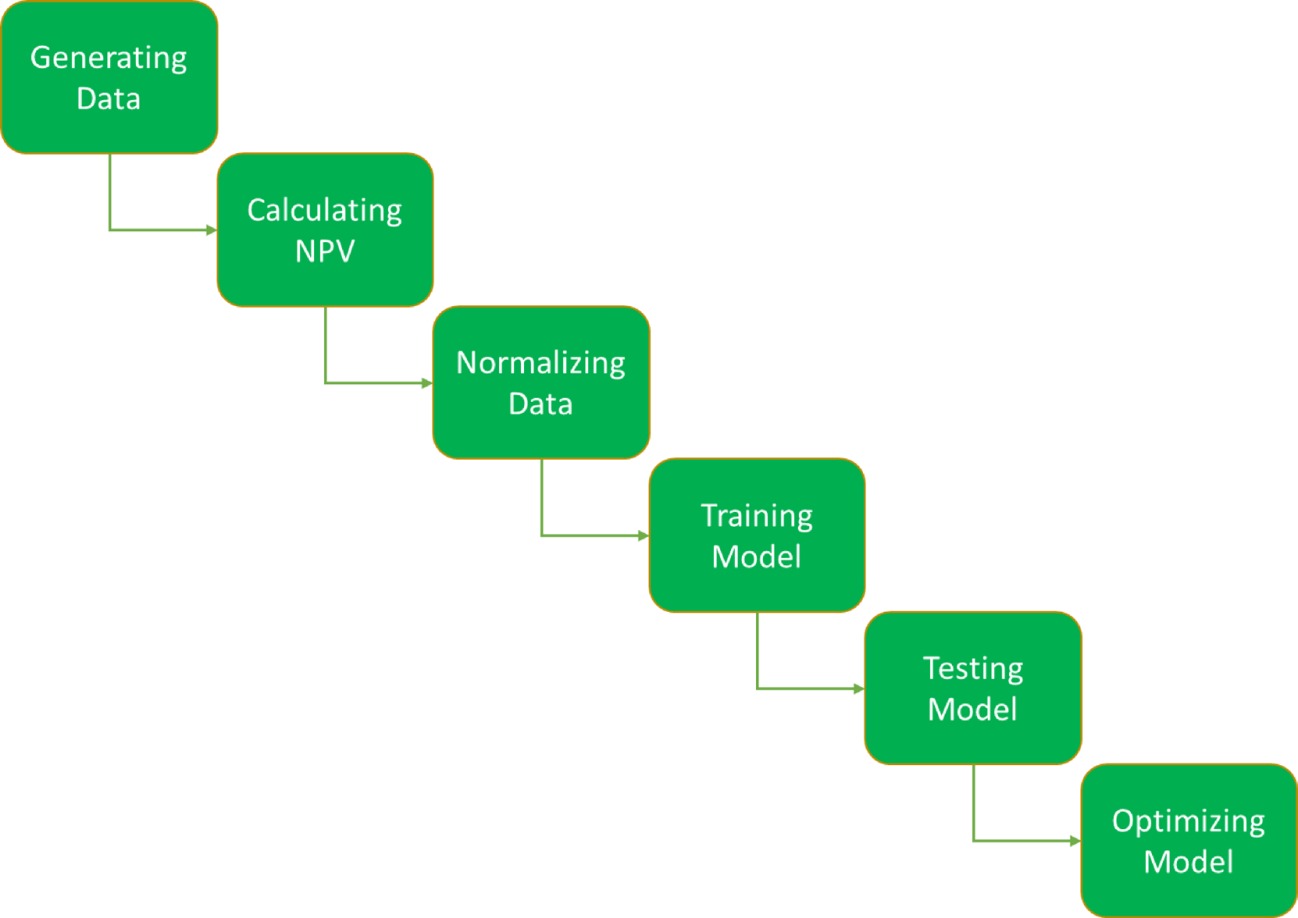
Workflow for water flooding optimization by proxy models.

In approach (1), we initially generate two groups of test states (each group 1024 states) employing the Latin hypercube sampling and Taguchi design of experiment technique and execute these states with the Eclipse E100 reservoir simulator software to compute the NPV value for each state. Subsequently, the resulting data are normalized to the maximum and minimum values. In the next stage, four proxy models are developed utilizing three deep neural network architectures (ANN, LSTM, GRU and EL), enabling the computation of the NPV value by inputting control parameters into the model. In this stage, 80% of the data was randomly selected to train the model, while the remaining 20% was utilized to evaluate the model. In the subsequent step, we optimize the well control parameters to maximize the NPV value by defining a cost function that produces the NPV value based on the well control parameters and applying the particle swarm optimization (PSO) and Bayesian optimization (BO) algorithms. Figure 2 shows the optimization workflow for the proxy models.

In method (2) for optimizing the well control parameters, a cost function is initially defined. The well control parameters are taken as inputs, and then the cumulative oil and water produced, as well as the cumulative water injected, are calculated using the Eclipse E100 reservoir simulator software. The NPV is generated, and subsequently, this cost function is optimized through the PSO and BO algorithm.

### Reservoir model

The PUNQ-S3 benchmark reservoir was selected for this study due to its wide recognition and availability in the reservoir engineering community, which facilitates reproducibility and benchmarking of new methodologies. While it is relatively simple in geological complexity, this characteristic allows for more straightforward interpretation of the performance differences between proxy and real reservoir models without the confounding influence of extreme heterogeneity or uncertainty. Moreover, modifications were introduced to the original PUNQ-S3 dataset in this study to simulate more realistic and challenging reservoir conditions, thereby enhancing its suitability for testing model robustness. These controlled modifications strike a balance between complexity and interpretability, providing a rigorous yet transparent platform for evaluating workflow reliability before scaling to more complex real-world reservoirs.

**Fig. 3:**
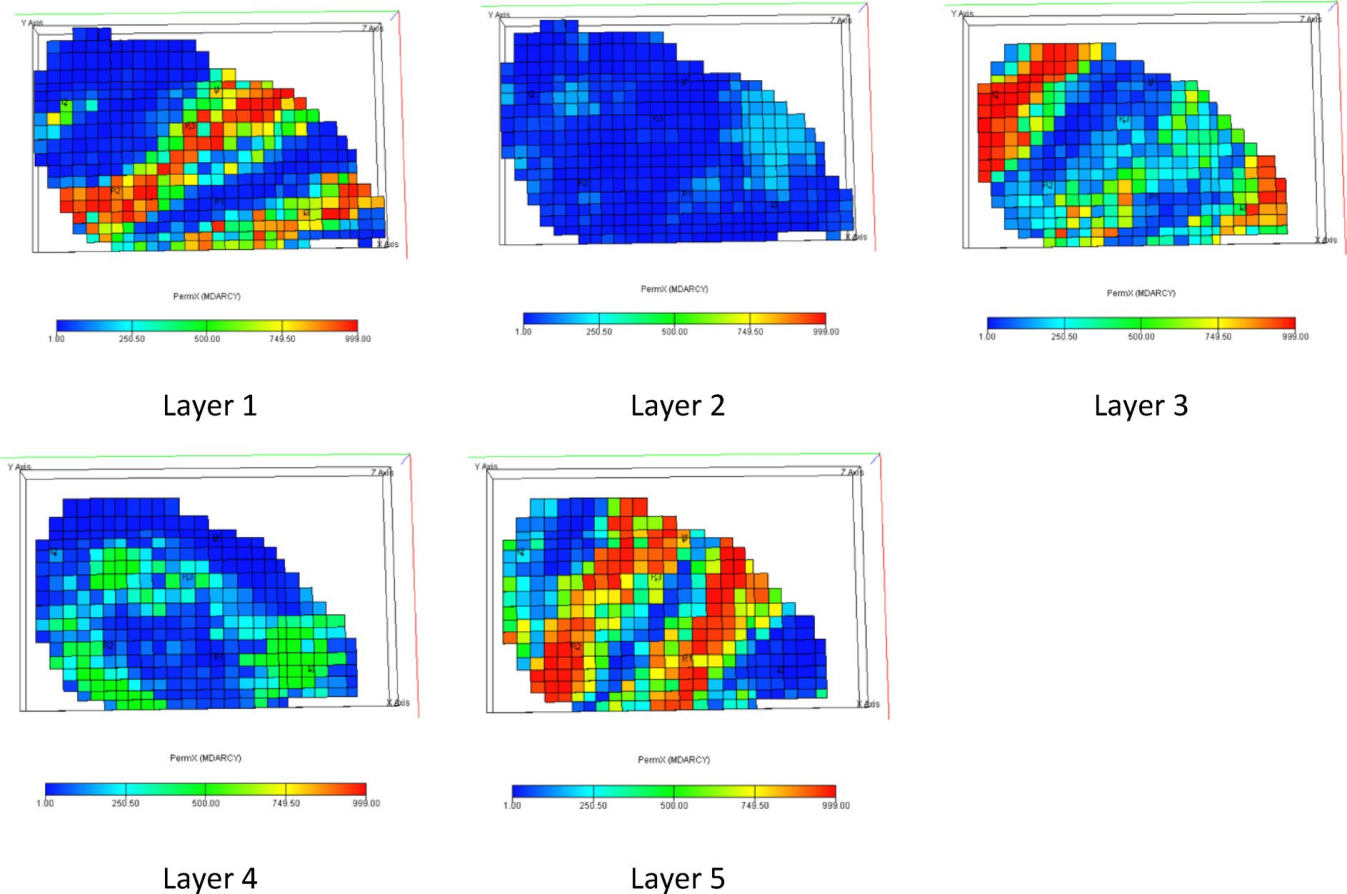
Permeability distribution in reservoir layers.

**Fig. 4:**
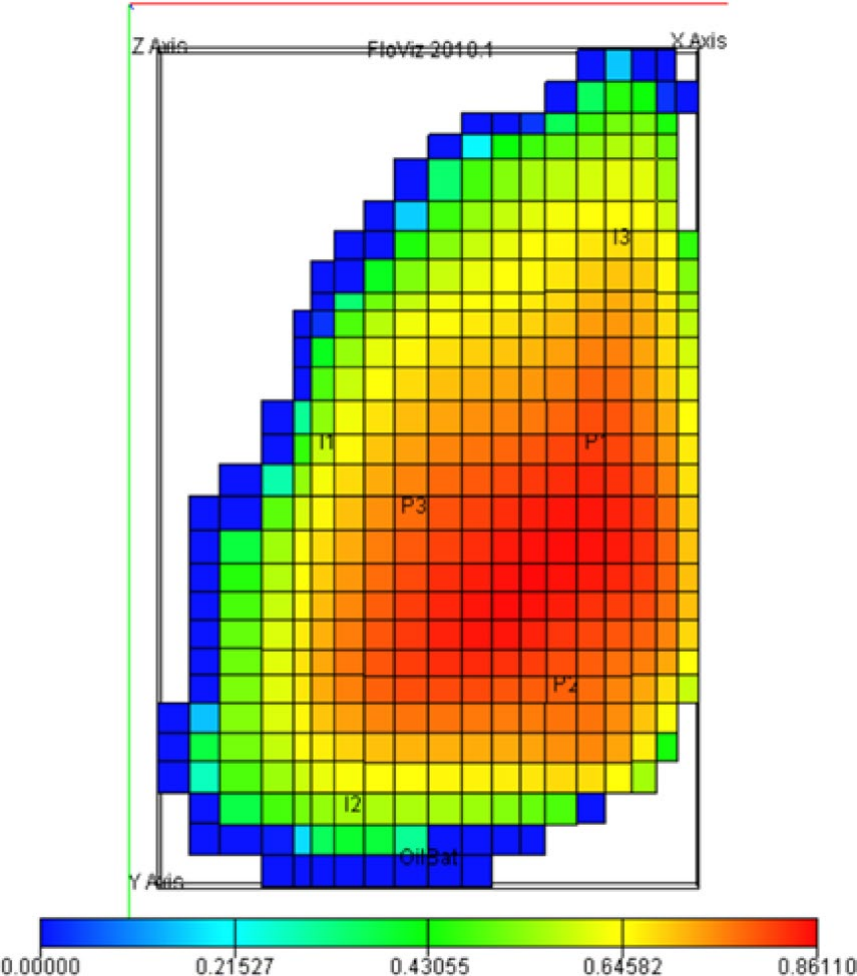
Oil saturation and location of wells in the PUNQ-S3 model.

This model consists of 5 layers at a depth of 2430 m and a slope of 1.5 degrees and includes 5 × 28 × 19 grid blocks, of which 1761 blocks are active and cover a uniform area of 180 × 180 square meters which the permeability distribution in these five layers is shown in Fig. 3. The top view of the reservoir structure which indicates oil saturation is shown in Fig. 4. The model consists of two phases of water and oil in terms of reservoir fluid properties, which are listed in Table 1.

Two faults are located in the east and south of the model, and the north and west are connected to a large active aquifer (Carter Tracy). Six wells have been drilled in the reservoir, half of which are production wells, and the rest are injection wells. All wells have penetrated all five layers of the model.

**Table 1:** Other properties of the benchmark reservoir model used.

Item	Parameter	Unit	Value
1	Initial reservoir pressure	psi	4840
2	Water/Oil contact(WOC)	ft.	7857
3	Oil density	pcf	56.93
4	Water density	pcf	62.428
5	Oil formation volume factor	resbbl/STB	1.06
6	Water formation volume factor	resbbl/STB	1.004
7	Oil viscosity	cp.	1.55
8	Water viscosity	cp.	0.5
9	Bubble point pressure	psi	1000

### Net present value calculation

#### Objective function

The objective is to maximize the NPV of the cumulative oil and water production over a fixed period. The objective function (or cost function) is thus given by following Eq^39^.:

1 $$NPV=\sum\limits_{{k=1}}^{K} {\left[ {({r_o}{q_{o,k}} - {r_{wp}}{q_{wp,k}} - {r_{wi}}{q_{wi,k}}).\Delta {t_k}} \right]}$$

Where:

- r_o_​: oil price ($/STB).
- r_wp_​: water production cost ($/STB).
- r_wi_: water injection cost ($/STB).
- q_o, k_​: oil production rate at time step k.
- q_wp, k_: water production rate at time step k.
- q_wi, k_​: water injection rate at time step k.
- Δt_k_: duration of time step k.
- K: total number of time steps.

In this study, a zero discount rate (i.e., b = 0) is assumed, consistent with similar benchmarking studies, and to focus purely on operational performance without introducing economic uncertainty. Table 2 indicates oil price, water production cost, water injection cost which assumed in this study.

**Table 2:** NPV parameter values per barrel.

Oil price (ro)	70 $
Water production cost (rwp)	5 $
Water injection cost (rwi)	5 $

#### Decision variables

The decision variables in this optimization framework are:

- Injection rates for each injector well over time.
- Production rates (or bottom-hole pressures) for each producer well over time.

These control variables are selected because they directly influence reservoir dynamics, fluid movement, and ultimately, the efficiency and economics of the waterflooding process. By varying these inputs, the proxy and real reservoir models predict the resulting production profiles and associated economic outcomes (NPV), which are then optimized.

#### Constraints

The optimization is subject to the following physical and operational constraints:

- Minimum and maximum flow rates for production wells, and minimum and maximum injection pressure for injection wells, based on well design and reservoir capacity:

• $$1000STBD \leqslant {q_o} \leqslant 10000STBD$$.

• $$2000psi \leqslant {P_{wi}} \leqslant 8000psi$$.

- Reservoir pressure bounds to avoid exceeding fracture pressure or dropping below bubble point: maximum fracture pressure assumed 8000 psi, and minimum injection pressure considered 2000 psi to avoid pressure reduction under bobble point pressure.
- Operational constraints such as well shut-in criteria or surface facility limitations: maximum water cut for production wells determined 0.5.

These constraints ensure that the optimized strategies are not only theoretically optimal but also operationally feasible and physically realistic, which is essential for applicability in field scenarios.

### Design of experiment (DOE)

Any situation or setting that can change during the process is referred to as the independent variable, and the output or result of the process is called the dependent variable. Independent variables are typically referred to as control factors (X) and dependent variables (Y). Some factors such as temperature, and time can be continuously changed and other factors are discontinuous, such as the type of material, and the presence or absence of a step in the process. Control factors are systematically changed during the experiment, and their different values ​​are called levels. Each series of experiments that has its conditions is known as an experiment run. The most basic model of experiments, which is obtained by using any possible combination of control factors and levels, is called factorial experiments^40^.

#### Taguchi (T) method

The Taguchi Design of Experiment (DOE) method is a robust and efficient statistical approach developed by Dr. Genichi Taguchi to improve product quality and process performance by minimizing variability and identifying optimal conditions with a limited number of experiments^41^. Unlike traditional experimental designs, Taguchi’s method emphasizes the use of orthogonal arrays (OAs) to systematically and economically study the effects of multiple factors on performance outcomes^42^. This approach facilitates the investigation of a large number of variables with a reduced number of experimental runs, making it particularly suitable for complex industrial processes and engineering applications^43^.

#### Latin hypercube sampling (LHS) method

The Latin Hypercube Sampling (LHS) method is an efficient statistical technique used for the design of experiments and sampling in multidimensional parameter spaces. Proposed initially by McKay et al.^44^ LHS ensures that the entire range of each input variable is explored systematically, leading to a more comprehensive and representative sampling compared to simple random sampling. In LHS, the input space is divided into equally probable intervals, and samples are drawn in such a way that each interval for every variable is sampled exactly once. This stratified sampling approach minimizes the risk of clustering and reduces the variance of the simulation results, even with relatively small sample sizes^45^. Due to its efficiency in covering the design space, LHS is widely used in computer experiments, sensitivity analysis, uncertainty quantification, and surrogate modeling^46^. Furthermore, the method can be integrated with optimization techniques and machine learning models to enhance the accuracy and robustness of complex system analyses^47^.

In this research, the Taguchi and Latin hypercube sampling (LHS) design of experiment methods are employed to select sample runs for producing data sets. Through the application of these sampling methods, 1024 data points are produced from six well-control parameters to compute NPV. The choice of 1024 samples was based on achieving a balance between computational efficiency and sufficient coverage of the input parameter space, as recommended in previous studies on surrogate modeling and machine learning for reservoir simulation^48^. This sample size ensures statistical reliability while maintaining a manageable computational cost.

The six parameters consist of the production rates of production well #1 (R1), production well #2 (R2), production well #3 (R3), as well as the injection pressures of injection well #1 (P1), injection well #2 (P2), and injection well #3 (P3). Table 3 displays the data range taken into account for generating data points through Taguchi and LHS methods. Table 4 and.

Table 5 statistically analyze the data generated by the two experimental design methods, which show count, mean, standard deviation(std), minimum, first quartile(Q1), median, third quartile(Q3), and maximum of inputs and outputs data.

**Table 3:** Data range to generate data points.

	R1(STBD)	R2(STBD)	R3(STBD)	P1(psi)	P2(psi)	P3(psi)
Min	1000	1000	1000	2000	2000	2000
Max	10,000	10,000	10,000	8000	8000	8000

**Table 4:** Statistical summary of the LHS dataset.

Statistic	R1(STBD)	R2(STBD)	R3(STBD)	P1(psi)	P2(psi)	P3(psi)	NPV (Thousand $)
count	1024	1024	1024	1024	1024	1024	1024
mean	5499.93	5499.91	5499.95	4999.99	4999.91	4999.91	1127.91
std	2599.37	2599.26	2599.36	1732.86	1732.97	1732.93	243.52
min	1001.05	1002.09	1006.91	2001.77	2004.29	2001.21	397.89
Q1	3253.58	3254.11	3250.60	3501.26	3498.94	3502.96	969.71
median	5500.60	5500.86	5501.39	5000.23	4999.91	5001.54	1104.27
Q3	7748.36	7746.95	7750.55	6497.65	6498.16	6498.91	1320.47
max	9993.57	9994.98	9998.07	7997.35	7995.76	7995.73	1643.47

**Table 5:** Statistical summary of the Taguchi dataset.

Statistic	R1(STBD)	R2(STBD)	R3(STBD)	P1(psi)	P2(psi)	P3(psi)	NPV (Thousand $)
count	1024	1024	1024	1024	1024	1024	1024
mean	5640.62	5640.62	5640.62	5093.75	5093.75	5093.75	1383.50
std	2598.07	2598.07	2598.07	1732.05	1732.05	1732.05	271.68
min	1281.25	1281.25	1281.25	2187.5	2187.5	2187.5	697.25
Q1	3460.93	3460.93	3460.93	3640.62	3640.62	3640.62	1214.03
median	5640.62	5640.62	5640.62	5093.75	5093.75	5093.75	1360.08
Q3	7820.31	7820.31	7820.31	6546.87	6546.87	6546.87	1602.02
max	10,000	10,000	10,000	8000	8000	8000	1976.98

### Deep learning algorithms

In this study, to create the reservoir proxy model, four deep learning approaches were employed for the two types of datasets generated by the Taguchi and LHS methods, including: (1) Artificial Neural Network (ANN), (2) Long Short-Term Memory (LSTM), (3) Gated Recurrent Unit (GRU), and (4) Ensemble Learning(EL). ANN was selected for its strong capability in modeling nonlinear static relationships, while LSTM and GRU networks, although typically applied to time-series forecasting, were adapted here for static regression tasks by reshaping the static input features into artificial sequences. This adaptation enabled the models to exploit their gating mechanisms to capture complex nonlinear interactions among input parameters. Similar strategies have been applied in previous studies where sequential models were repurposed for static predictions (e.g., Zhang et al., 2021; Li et al., 2020), demonstrating potential benefits in uncovering subtle dependencies between features. Additionally, EL was employed to combine the outputs of ANN, LSTM, and GRU, thereby leveraging their complementary strengths. Ensemble Learning was employed to integrate the predictive power of ANN, LSTM, and GRU, reducing individual model limitations such as bias and variance. This approach improves generalization and enhances the accuracy of the proxy model. Consequently, with two types of datasets and four modeling approaches, a total of eight proxy models were developed for comprehensive performance evaluation.

#### Artificial neural network (ANN)

**Fig. 5:**
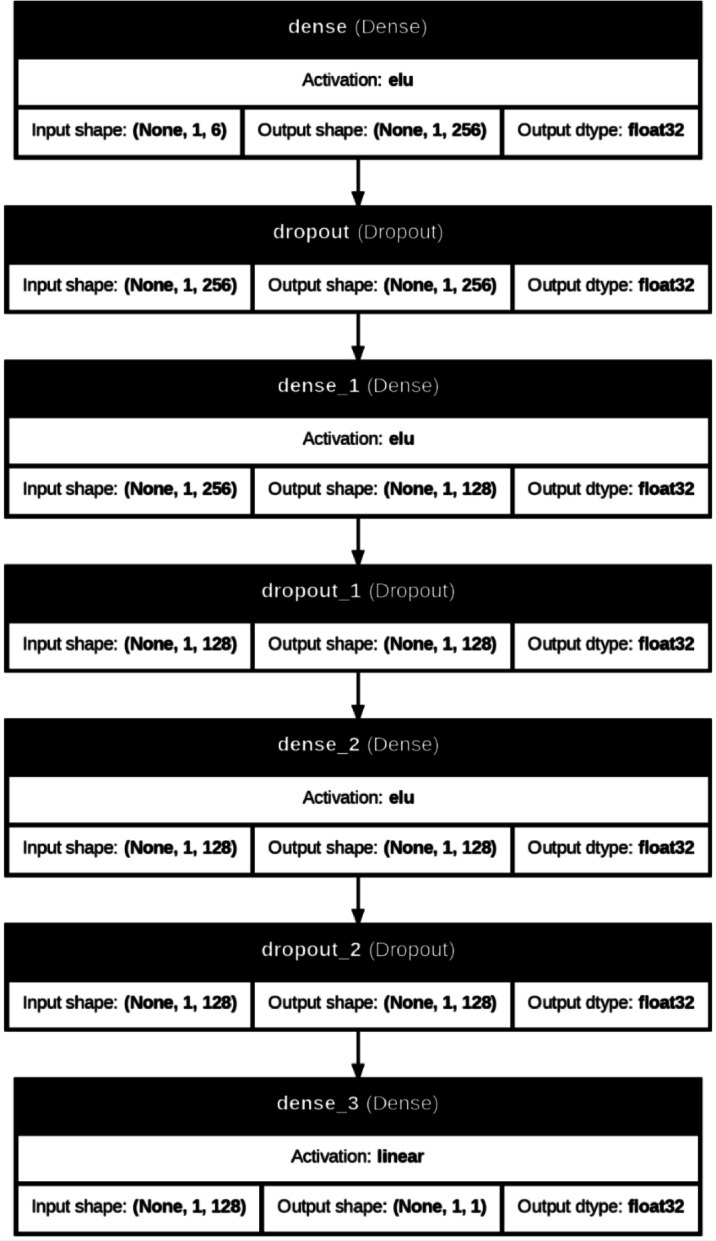
ANN network structure.

Artificial Neural Networks (ANNs) are a class of machine learning algorithms inspired by the structure and functioning of the human brain. They are capable of modeling complex, non-linear relationships in data and have been successfully applied in various domains such as classification, regression, pattern recognition, and forecasting tasks^49,50^. An ANN consists of interconnected processing units, known as neurons, organized into layers—typically including an input layer, one or more hidden layers, and an output layer. Each neuron processes weighted inputs and applies an activation function to produce an output, enabling the network to learn from data through an iterative training process^51^. The learning process in ANNs generally employs supervised learning methods with algorithms like backpropagation and optimization techniques such as stochastic gradient descent (SGD) or Adam optimizer to minimize prediction errors^52^. Due to their ability to automatically extract features and generalize from large datasets, ANNs have become a powerful tool in solving complex real-world problems across multiple disciplines^53^. Figure 5 shows the structure of the ANN used in this study, indicating the layers, nodes, and activation functions.

#### Long short-term memory (LSTM)

LSTM is a special type of recurrent neural network that can learn long-term data dependencies. Apart from the output of the previous time step, there is another relationship between the units called cell state. This connection acts as an easy path for information flow between units. Only minor linear interactions occur for the cellular state. So it is very easy for information to flow without change. Information is added to the cell state with the help of gates. The gates are a neural network layer with an activation function. Thus, a value of one means that all information is transferred to the cell states at the current time, while a value of zero means that nothing is transferred to the cell state^54^.

**Fig. 6:**
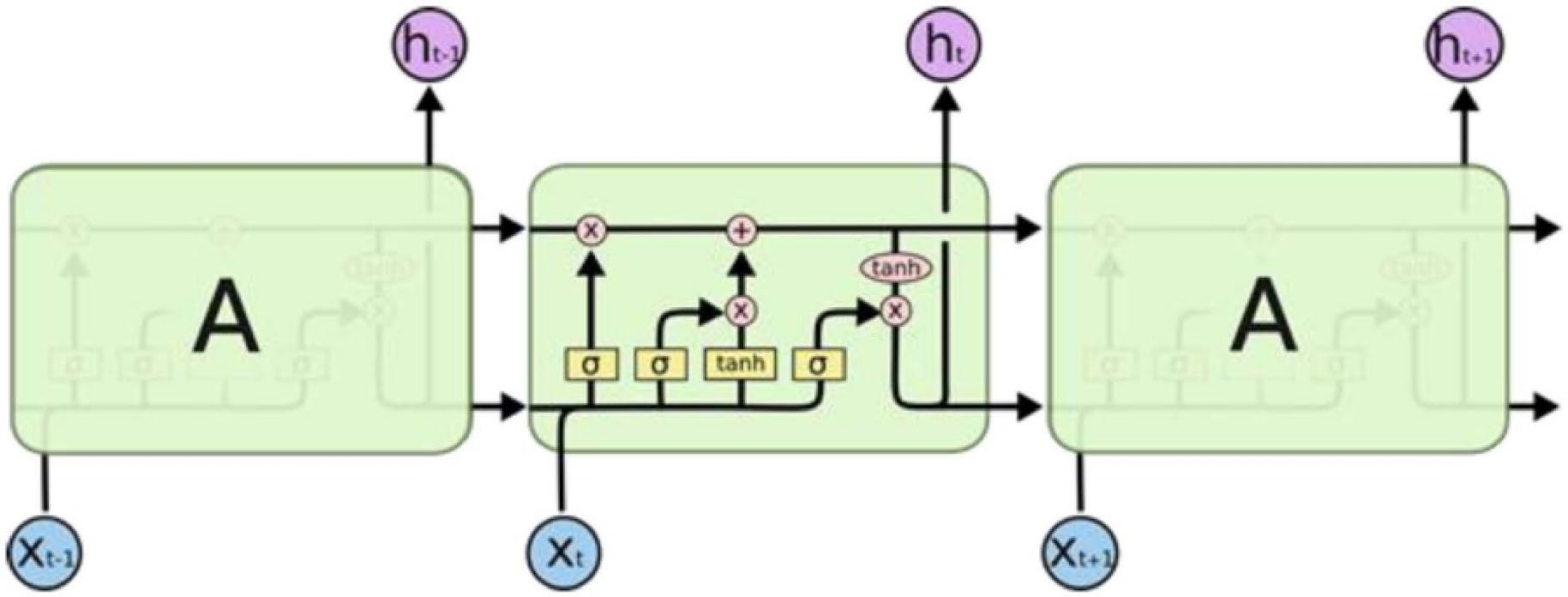
Architecture of LSTM^55^.

**Fig. 7:**
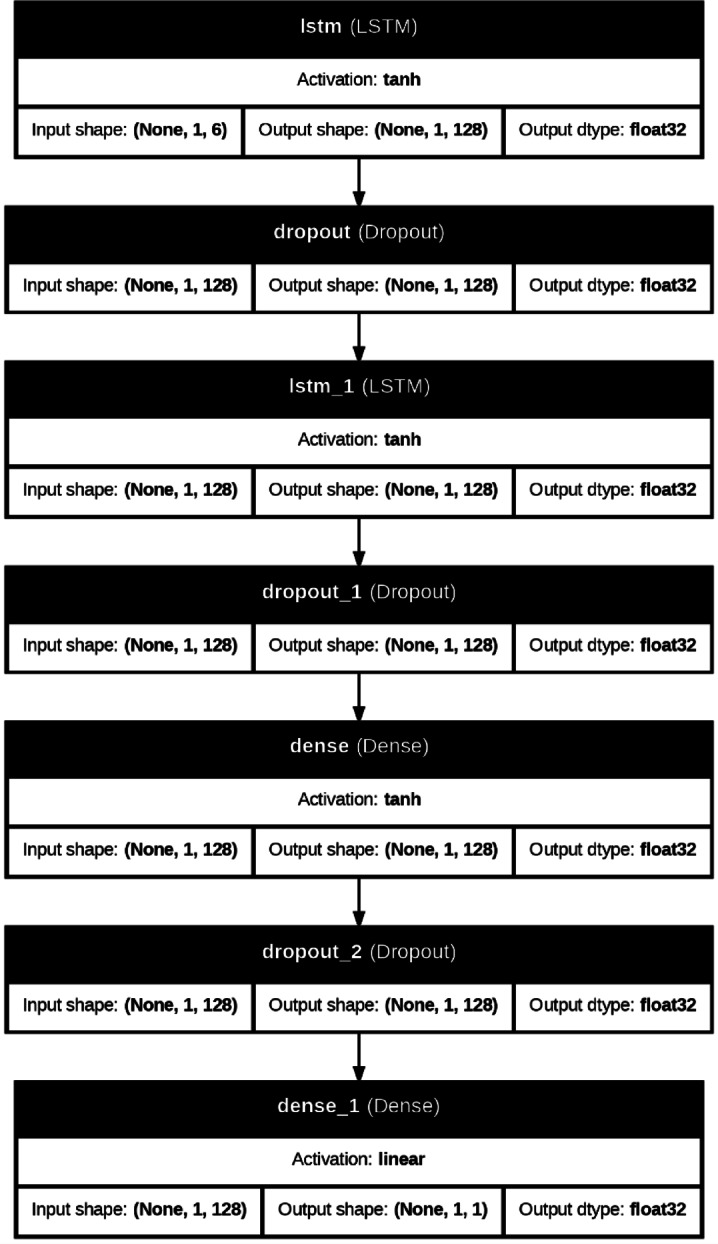
LSTM network structure.

LSTM has many real-world applications. It is suitable for classification, processing, and prediction problems. It is used in language translation, text auto-completion, speech recognition, handwriting recognition, anomaly detection, and recently for time series data prediction^54^. Figure 6 shows the LSTM architecture, and Fig. 7 indicates the LSTM network structure utilized in this research.

#### Gated recurrent unit (GRU)

**Fig. 8:**
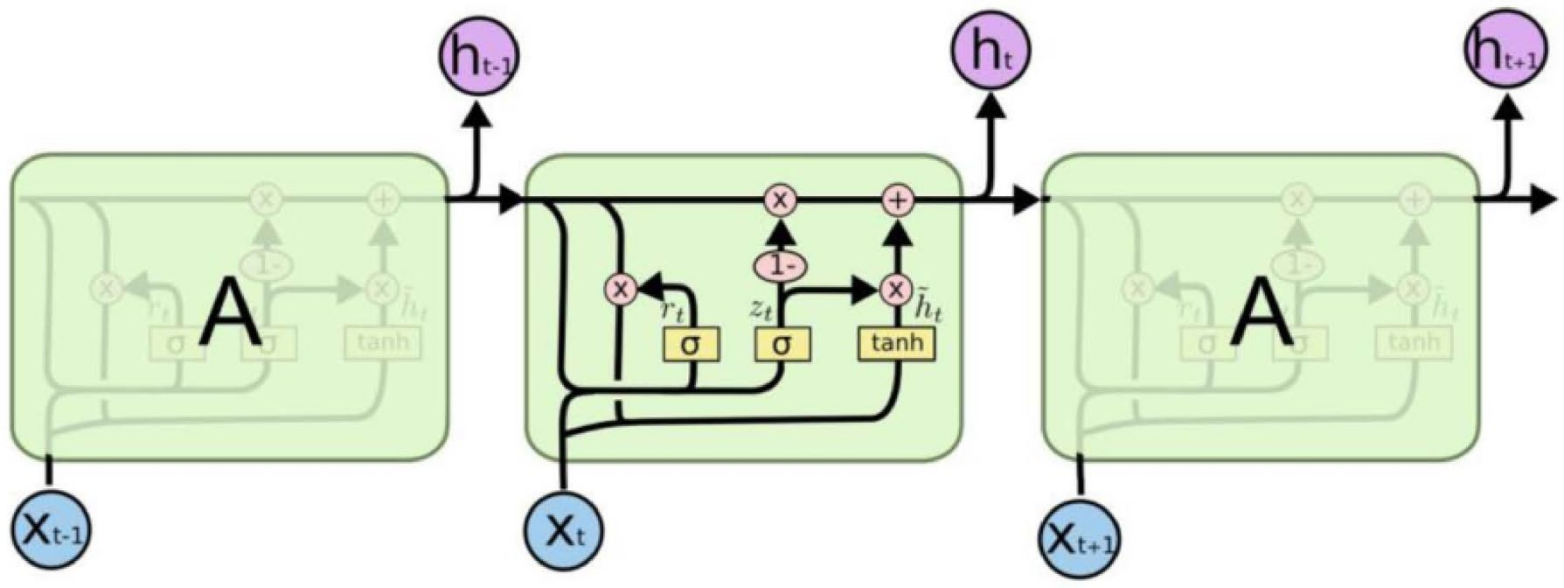
Architecture of GRU^55^.

**Fig. 9:**
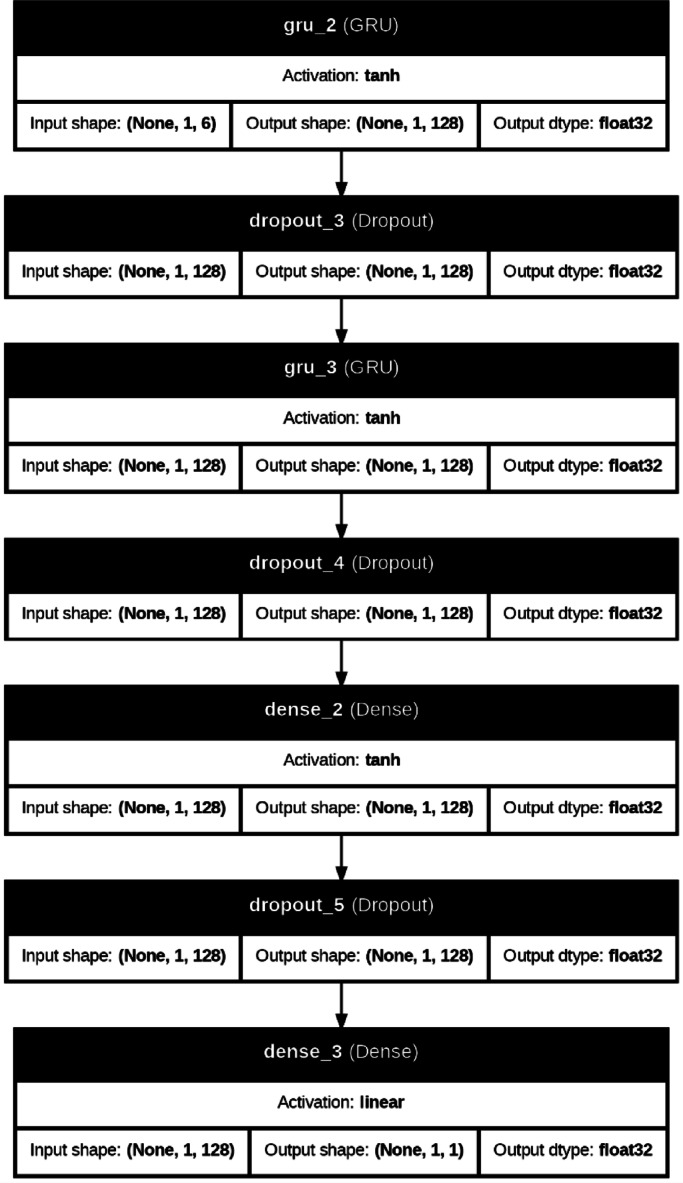
GRU network structure.

A gated recurrent unit is a simplified variant of LSTM. Figure 8 shows the GRU network architecture. GRU has a more cost-effective architecture than the LSTM network. GRU has fewer parameters than LSTM for a given problem. LSTM has two connections between cells (previous output and cell state memory). In contrast, GRU has only one connection between cells and has two gates under the headings of update gate and forget gate^56^. Figure 9 indicates the GRU network structure utilized in this paper.

#### Ensemble learning (EL)

Ensemble learning is a machine learning approach that combines multiple models to improve prediction accuracy, reduce variance, and enhance robustness compared to individual learners^57,58^. In this study, we developed an ensemble model using the last three neural architectures—Artificial Neural Networks (ANN), Long Short-Term Memory (LSTM), and Gated Recurrent Unit (GRU). Here, each model contributes to the final prediction, ensuring balanced representation of their complementary strengths in nonlinear approximation and sequence modeling.

**Fig. 10:**
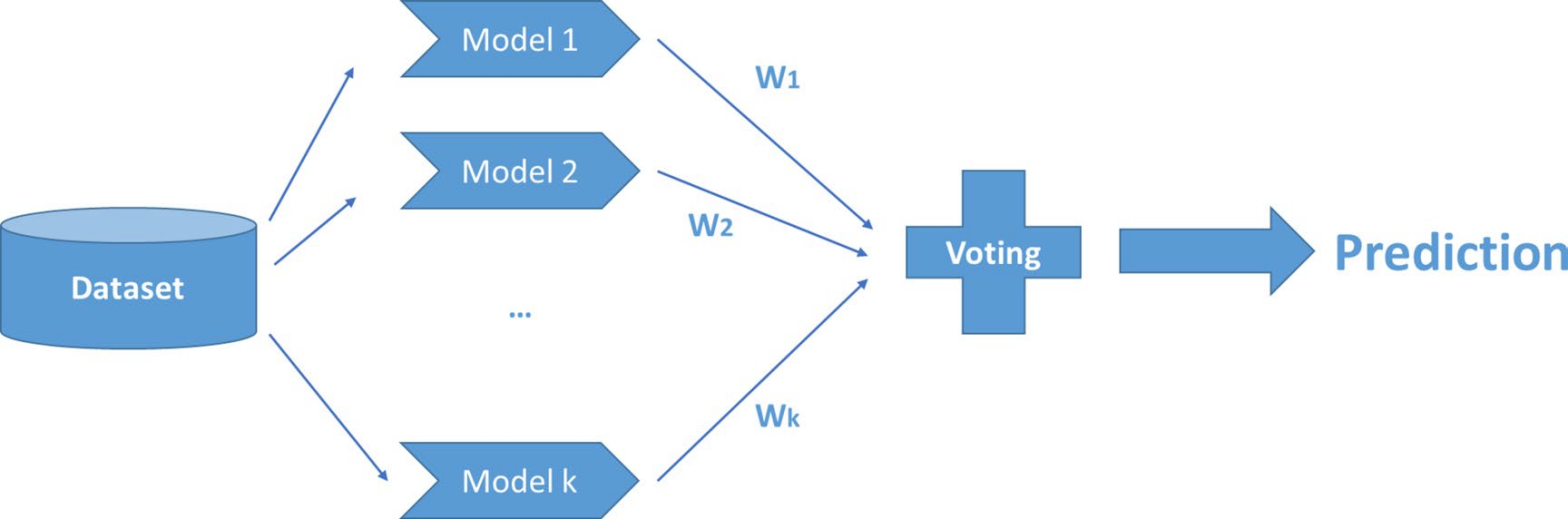
Voting involves training multiple base models on the entire dataset and constructing an ensemble whose outcome represents an average of the predictions made by these learners.

Voting regression represents commonly applied ensemble technique. It integrates diverse regression models and produces predictions by averaging their outputs. The strategy used to merge these predictions is critical to the final performance^59^. As illustrated in Fig. 10, each base learner is assigned a specific weight in the ensemble.

In this study, it was used RRMSE (Relative Root Mean Squared Error) voting regressor as voting regression, which is defined as follow^59^:

2 $$RRMSE=\sqrt {\frac{{\frac{1}{n}\sum\limits_{{i=1}}^{n} {{{({x_{mi}} - {x_{pi}})}^2}} }}{{\sum\limits_{{i=1}}^{n} {{x_{pi}}^{2}} }}} *100\%$$

Where n represents the number of observations, $\:{x}_{m}$, and $\:{x}_{p}$ define the measured and predicted parameters. Then, it can be calculated weight for each model (w_i_) as follow:

3 $${w_i}=\frac{{\frac{1}{{RRMS{E_i}}}}}{{\sum\limits_{{i=1}}^{n} {\frac{1}{{RRMS{E_i}}}} }}$$

Assuming that , , and denote the input variables, the output variables, and the model function, respectively, ensemble learning can be expressed as follows:

4 $$Y=\sum\limits_{{i=1}}^{n} {{w_i}{f_i}(X)}$$

### Optimization algorithms

Optimization techniques are widely used to enhance model performance and ensure efficient parameter selection in complex problems. In this study, two approaches were employed: Particle Swarm Optimization (PSO), a population-based metaheuristic inspired by swarm intelligence^60^ and Bayesian Optimization, a probabilistic framework for efficient hyperparameter tuning^61^.

#### Particle swarm optimization (PSO)

Particle swarm optimization is an algorithm that simulates the behavior of a flock of birds^62^. For applications involving real decision variables, PSO emerged as a widely used optimization tool^63^. A particle moves in a multidimensional search space to find a possible response to a problem. Each particle alters its position depending on its experience and neighbors. The i-th particle in n-dimensional search space is presented as follows^64^:

5 $${X_i}=({x_{i1}},{x_{i2}},...,{x_{iN}})$$

The best among all the particles in the population$({P_g})$, the best previous location of each particle $({P_i})$, the velocity $({V_i})$, and the maximum velocity $({V_{\hbox{max} - i}})$ of each particle are expressed as^64^

6 $${P_i}=({p_{i1}},{p_{i2}},...,{p_{iN}})$$

7 $${P_g}=({p_{g1}},{p_{g2}},...,{p_{gN}})$$

8 $${V_i}=({v_{i1}},{v_{i2}},...,{v_{iN}})$$

9 $${V_{\hbox{max} - i}}=({v_{\hbox{max} - i1}},{v_{\hbox{max} - }}_{{i2}},...,{v_{\hbox{max} - }}_{{iN}})$$

Particles are limited by the maximum velocity set by the user. A maximum velocity encourages global exploration, while a low maximum velocity encourages local exploration^63^. An appropriate value must be specified to achieve a balance between these two states. The following relations^64^ can be utilized to determine the new particle’s velocity and position:

10 $${v_{i,n}}=w.{v_{i,n}}+{c_1}.{r_1}.({p_{i,n}} - {x_{i,n}})+{c_2}.{r_2}.({p_{g,n}} - {x_{g,n}})$$

11 $${x_{i,n}}={x_{i,n}}+{v_{i,n}}$$

Acceleration constants c_1_, c_2_, and inertia weight w are used in the above relations assumed 0.5, 0.3, and 0.9, respectively. Particles are driven toward their best positions based on these values. Furthermore, r_1_ and r_2_ are uniformly generated random numbers between 0 and 1 ^64^. Current velocity is influenced by previous velocity history through the inertia weight^65^. A constriction parameter is defined in some problems to enhance PSO performance and its ability to control particle velocity^64,66^:

12 $$K=\frac{2}{{\left| {2 - \varphi - \sqrt {{\varphi ^2} - 4\varphi } } \right|}}$$

Therefore;

13 $$\varphi ={\varphi _1}+{\varphi _2}(\varphi>4)$$

#### Bayesian optimization (BO)

Bayesian optimization (BO) is a global optimization technique well suited for problems where the objective function is expensive to evaluate, non-convex, and derivative-free^67^. Unlike population-based algorithms such as PSO, Bayesian optimization constructs a probabilistic surrogate model of the objective function and uses it to guide the search process efficiently^68^.

The general optimization problem can be expressed as^69^:

14 $$y=f(x),x \in \chi \subset {{\mathbb{R}}^d}$$

Where x represents a d-dimensional decision vector and y is the black-box response. Because direct evaluations of $f(x)$ are costly, BO employs a Gaussian Process (GP) as a surrogate^70^:

15 $$f(x)\sim GP(m(x),k(x,x\prime ))$$

Where $m(x)$ is the mean function and $k(x,x^{\prime})$ is a positive-definite covariance kernel. Given prior observations${D_t}=\{ ({x_i},{y_i})\} _{{i=1}}^{t}$, the GP posterior at a new candidate x is normally distributed^70^:

16 $$f(x)\mid {D_t}\sim N({\mu _t}(x),\sigma _{t}^{2}(x))$$

Here, ${\mu _t}(x)$ and $\sigma _{t}^{2}(x)$ denote the predictive mean and variance, respectively.

To select the next candidate point, BO maximizes an acquisition function $\alpha (x)$ that balances exploration (sampling uncertain regions) and exploitation (refining around known good areas). Common acquisition functions include:

1. Probability of Improvement (PI)^71^:

17 $${a_{PI}}(x)=\Phi (\gamma (x)),\gamma (x)=\frac{{f({x_{best}}) - \mu (x)}}{{\sigma (x)}}$$

Where $f({x_{best}})$ is the best current value, and$\Phi ( \cdot )$is the standard normal CDF.

2. Expected Improvement (EI)^72^:

18 $${a_{EI}}(x)=\sigma (x)[\gamma (x)\Phi (\gamma (x))+\phi (\gamma (x))]$$

Where$\Phi ( \cdot )$is the standard normal PDF. EI is widely used due to its closed form and effectiveness^67^.

3. Upper/Lower Confidence Bound (UCB/LCB)^73^:

19 $${a_{LCB}}(x)=\mu (x) - \kappa \sigma (x)$$

Where is a tunable parameter that directly controls the trade-off between exploration and exploitation. The next query point is obtained by maximizing the acquisition^73^:

20 $${x_{t+1}}=\mathop {argmax}\limits_{{x \in \chi }} \alpha (x)$$

### Model performance evaluation

It is necessary to recognize the criteria associated with evaluating model performance. In this work, root mean squared error (RMSE), mean absolute error (MAE), mean absolute percentage error (MAPE), R-squared (R^2^, and relative error were used as statistical indicators to evaluate the performance of the models, because they collectively provide a comprehensive evaluation of the model’s accuracy and predictive capability. RMSE and MAE measure the average magnitude of prediction errors, with RMSE giving more weight to larger errors, while MAPE expresses errors as a percentage, allowing for scale-independent comparison. R² indicates the proportion of variance explained by the model, reflecting its overall goodness-of-fit.

#### Root mean squared error (RMSE)

The root mean squared error is used to see how well the network output matches the desired output. Better performance is guaranteed with smaller RMSE values. It is defined as follows^74^:

21 $$RMSE=\sqrt {\frac{1}{n}\sum\limits_{{i=1}}^{n} {{{({x_{mi}} - {x_{pi}})}^2}} }$$

#### Mean absolute error (MAE)

The mean absolute error is the average value of the absolute difference between the predicted value and the actual value. Errors showing a uniform distribution shall be presented. Furthermore, MAE is the most natural and accurate measure of the average level of error^75^.

22 $$MAE=\frac{1}{n}\sum\limits_{{i=1}}^{n} {\left| {{x_{pi}} - {x_{mi}}} \right|}$$

#### Mean absolute percentage error (MAPE)

The mean absolute percentage error is calculated by dividing the absolute error of each period by the observed values ​​evident in that period. Then, average these fixed percentages. This approach is practical when the size or dimensions of the predictor variable are important in assessing the accuracy of the prediction^76,77^. MAPE indicates the degree of forecast error compared to the actual value.

23 $$MAPE=\frac{1}{n}\sum\limits_{{i=1}}^{n} {\frac{{\left| {{x_p}_{i} - {x_{mi}}} \right|}}{{{x_{mi}}}}} \times 100\%$$

#### R-squared (R^2^

An important index to check the correctness of the regression algorithm is$\:\:{R}^{2}$, which ranges from 0 to 1. $\:{R}^{2}$ is defined as follows^75^:

24 $${R^2}=1 - \frac{{\sum\limits_{{i=1}}^{n} {{{({x_{pi}} - {{\overline {x} }_m})}^2}} }}{{\sum\limits_{{i=1}}^{n} {{{({x_m}_{i} - {{\overline {x} }_m})}^2}} }}$$

Where n represents the number of observations, $\:{x}_{m}$, and $\:{x}_{p}$ define the measured and predicted parameters, respectively, and $\:\stackrel{-}{x}$_m_ signifies the average of measured parameters.

#### Relative error (RE)

The relative error is defined as the ratio of the difference of the predicted to the measured value. If $\:{x}_{m}$ is the measured value of a quantity, $\:{x}_{p}$ is the predicted value of the quantity, then the relative error can be measured using the below formula^78^.

25 $$RE=\frac{{{x_p} - {x_m}}}{{{x_m}}} \times 100\%$$

## Results and discussion

Given that the computational expense of actual reservoir models is quite significant and even utilizing computers with robust processors does not resolve this, reservoir engineers tend to generate several run modes. They employ neural networks and machine learning algorithms to develop a model referred to as a proxy model, allowing them to take advantage of these low-cost models instead of relying on real models that incur high computational expenses.

### Proxy models evaluation

#### ANN models

**Fig. 11:**
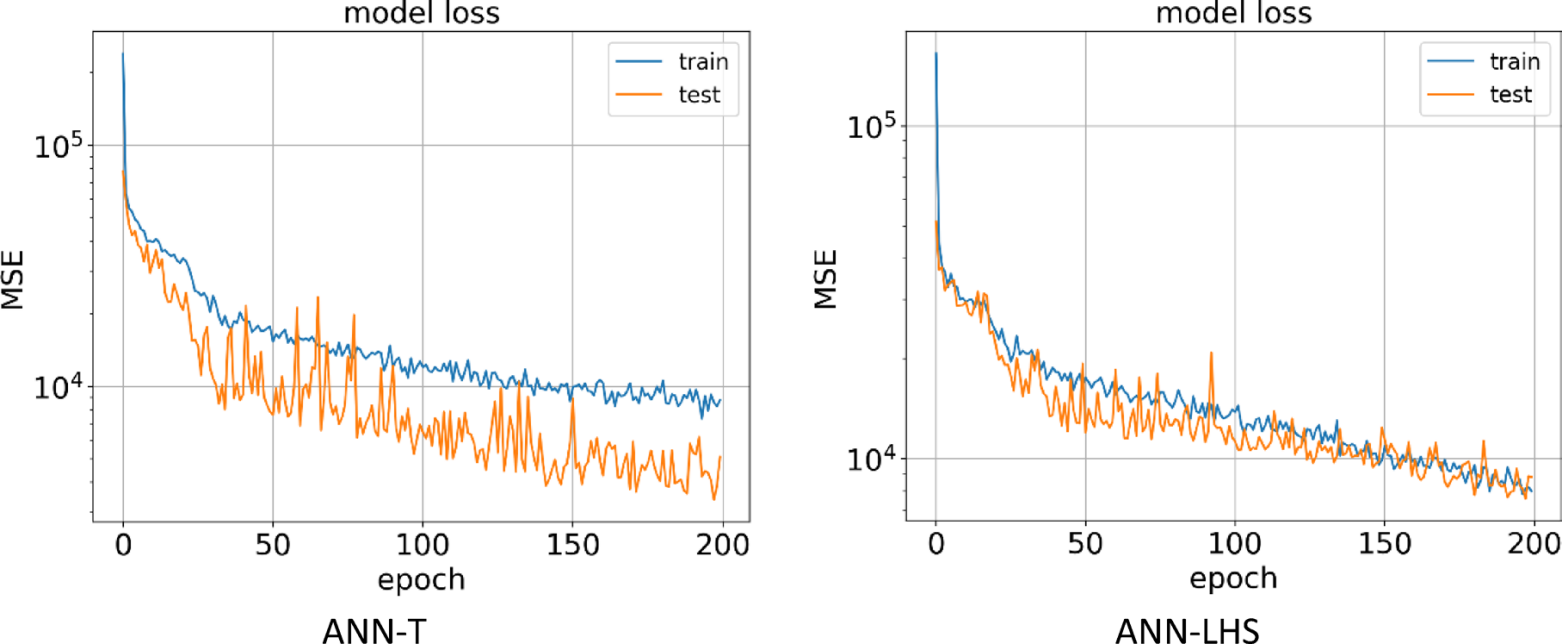
Train and test model loss vs. epochs obtained by ANN-T & ANN-LHS.

The first method for building a proxy was to use the ANN algorithm, which was performed on two datasets obtained from the LHS and Taguchi design of experiment methods. In Fig. 11, it can be seen the learning process of the AI ​​model in 200 epochs for the training and testing data for two types of datasets.

**Fig. 12:**
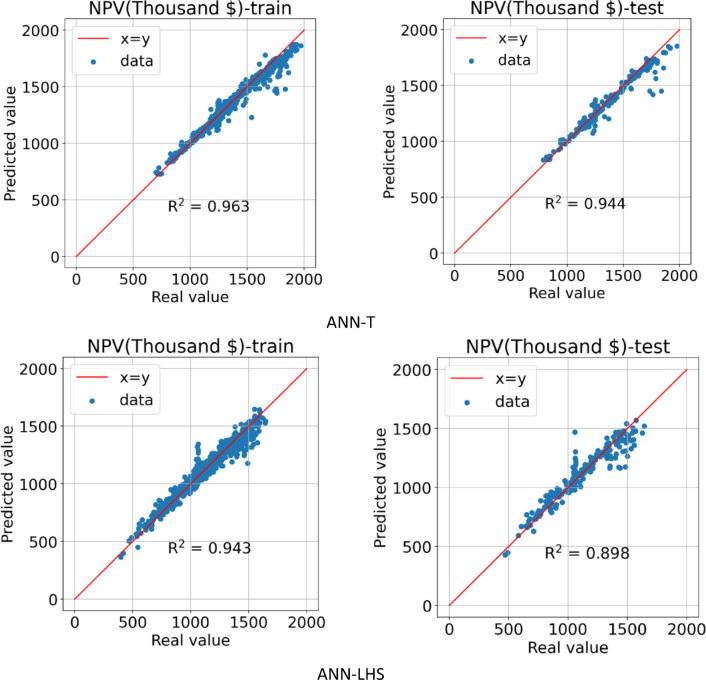
Predicted vs. real value for training and testing data obtained by ANN-T & ANN-LHS.

After the AI ​​model learning process is complete, Fig. 12 shows the predicted NPV value in terms of the actual value for the training and testing data for the ANN-LHS and ANN-T proxies. In the ANN-T proxy, the R^2^ values for the training and testing data are calculated and reported as 0.963 and 0.944, respectively. For the ANN-LHS proxy, the R^2^ values for the training and testing data are reported as 0.943 and 0.898, respectively, which indicates that the model accuracy was higher for the data obtained from the Taguchi design of experiment method.

#### GRU models

**Fig. 13:**
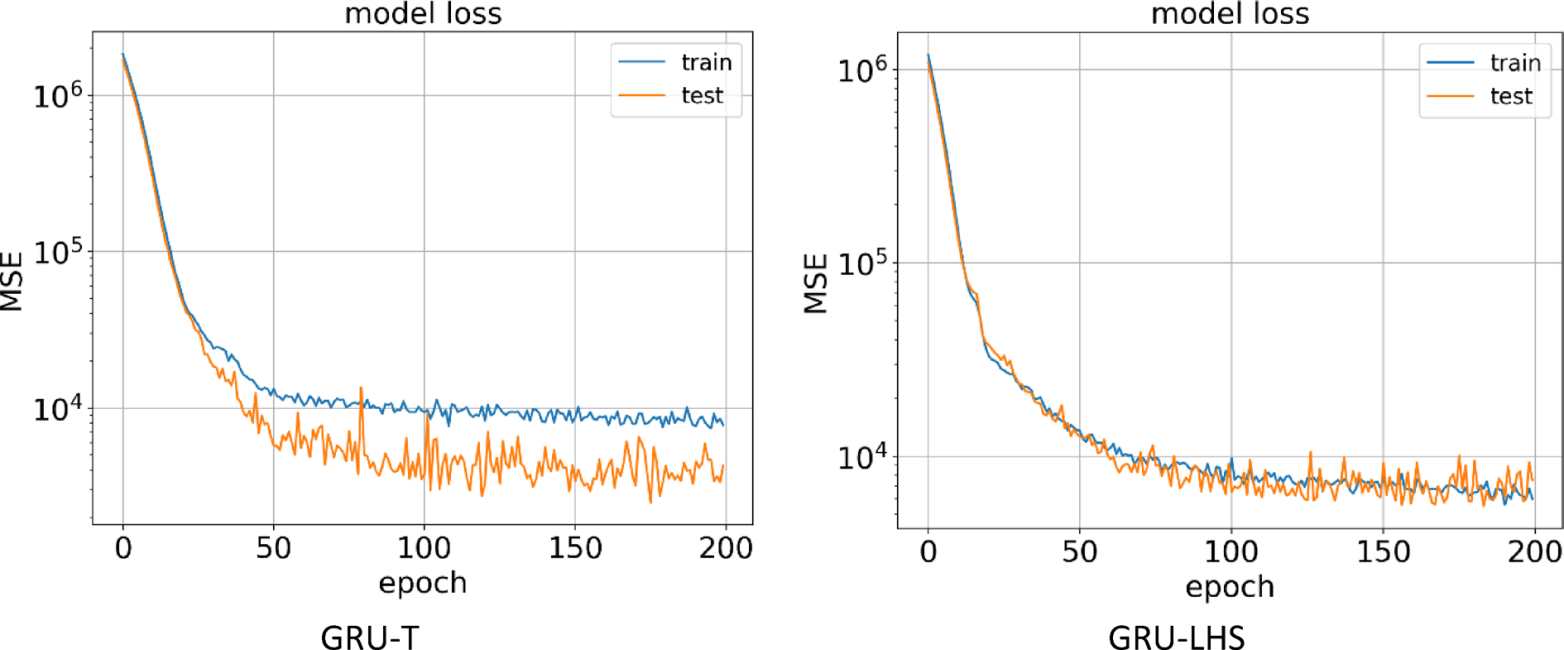
Train and test model loss vs. epochs obtained by GRU-T & GRU-LHS.

The second method for building a proxy was to use the GRU algorithm, which was performed on two datasets obtained from the LHS and Taguchi design of experiment methods. In Fig. 13, it can be seen the learning process of the AI ​​model in 200 epochs for the training and testing data for two types of datasets.

**Fig. 14:**
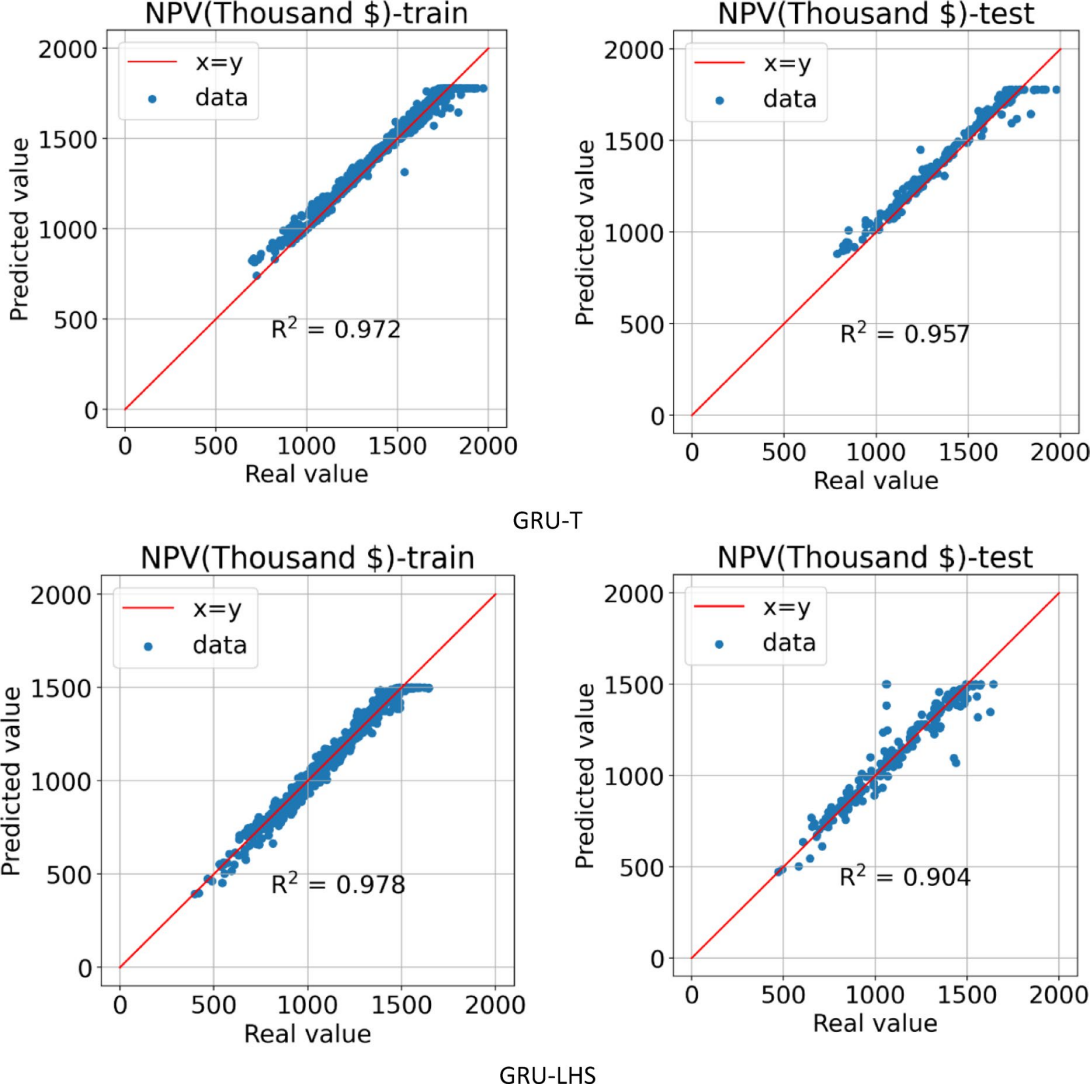
Predicted vs. real value for training and testing data obtained by GRU-T & GRU-LHS.

After the AI ​​model learning process is complete, Fig. 14 shows the predicted NPV value in terms of the actual value for the training and testing data for the GRU-LHS and GRU-T proxies. In the GRU-T proxy, the R^2^ values for the training and testing data are calculated and reported as 0.972 and 0.957, respectively. For the GRU-LHS proxy, the R^2^ values for the training and testing data are reported as 0.978 and 0.904, respectively, which indicates that the model accuracy was higher for the data obtained from the Taguchi design of experiment method.

#### LSTM models

**Fig. 15:**
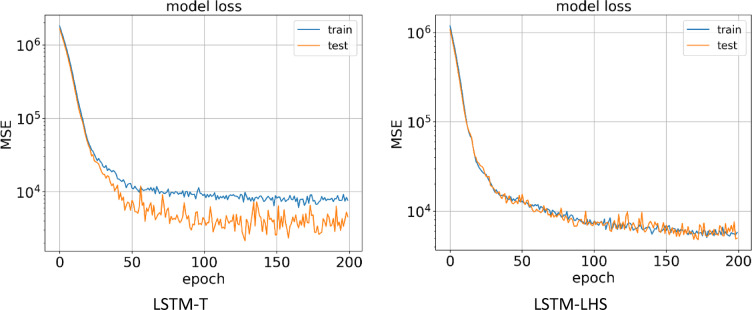
Train and test model loss vs. epochs obtained by LSTM-T & LSTM-LHS.

The third method for building a proxy was to use the LSTM algorithm, which was performed on two datasets obtained from the LHS and Taguchi design of experiment methods. In Fig. 15, it can be seen the learning process of the AI ​​model in 200 epochs for the training and testing data for two types of datasets.

**Fig. 16:**
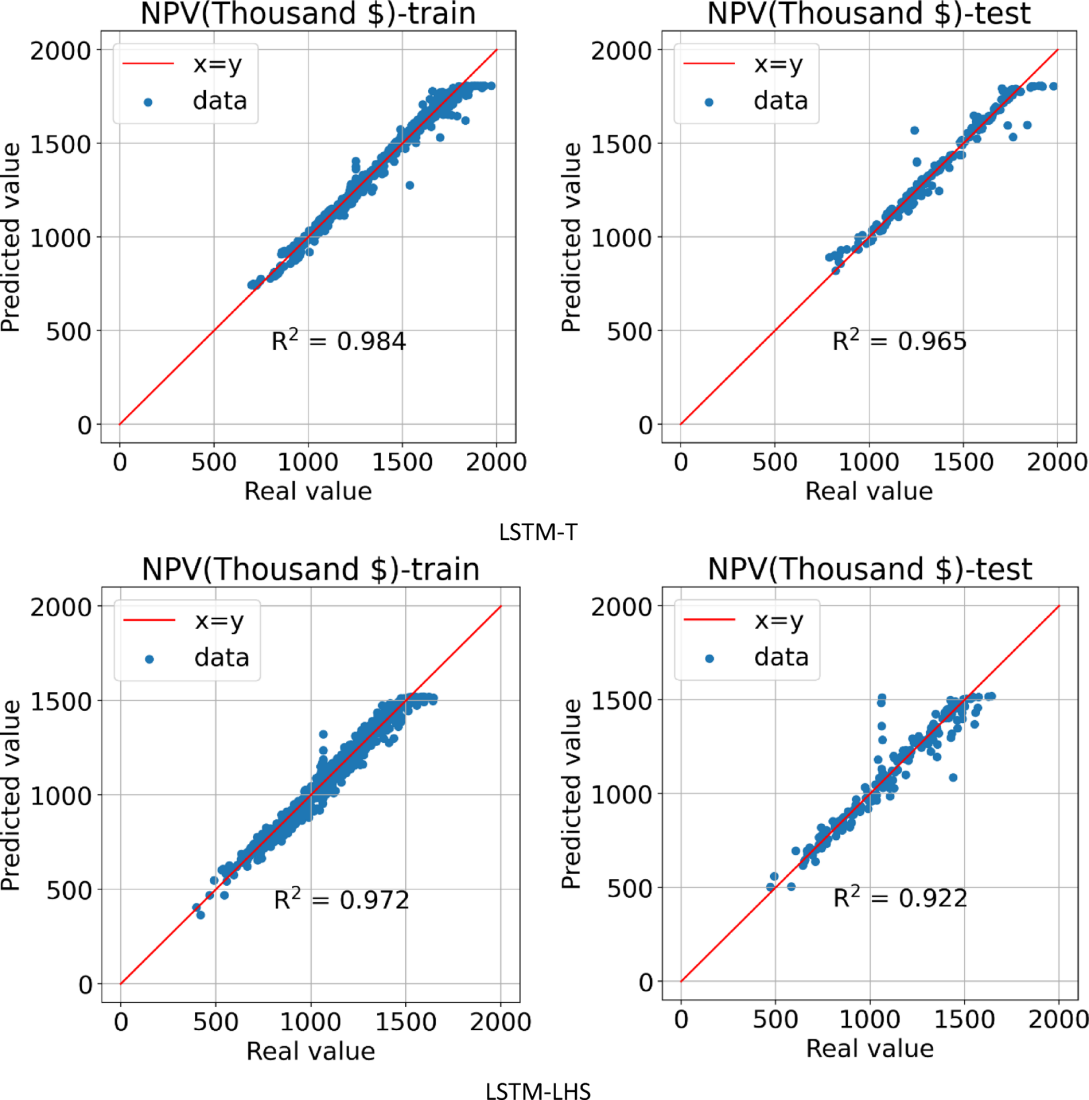
Predicted vs. real value for training and testing data obtained by LSTM-T & LSTM-LHS.

After the AI ​​model learning process is complete, Fig. 16 shows the predicted NPV value in terms of the actual value for the training and testing data for the LSTM-LHS and LSTM-T proxies. In the LSTM-T proxy, the R^2^ values for the training and testing data are calculated and reported as 0.984 and 0.965, respectively. For the LSTM-LHS proxy, the R^2^ value for the training and testing data are reported as 0.972 and 0.922, respectively, which indicates that the model accuracy was higher for the data obtained from the Taguchi design of experiment method.

#### EL models

**Fig. 17:**
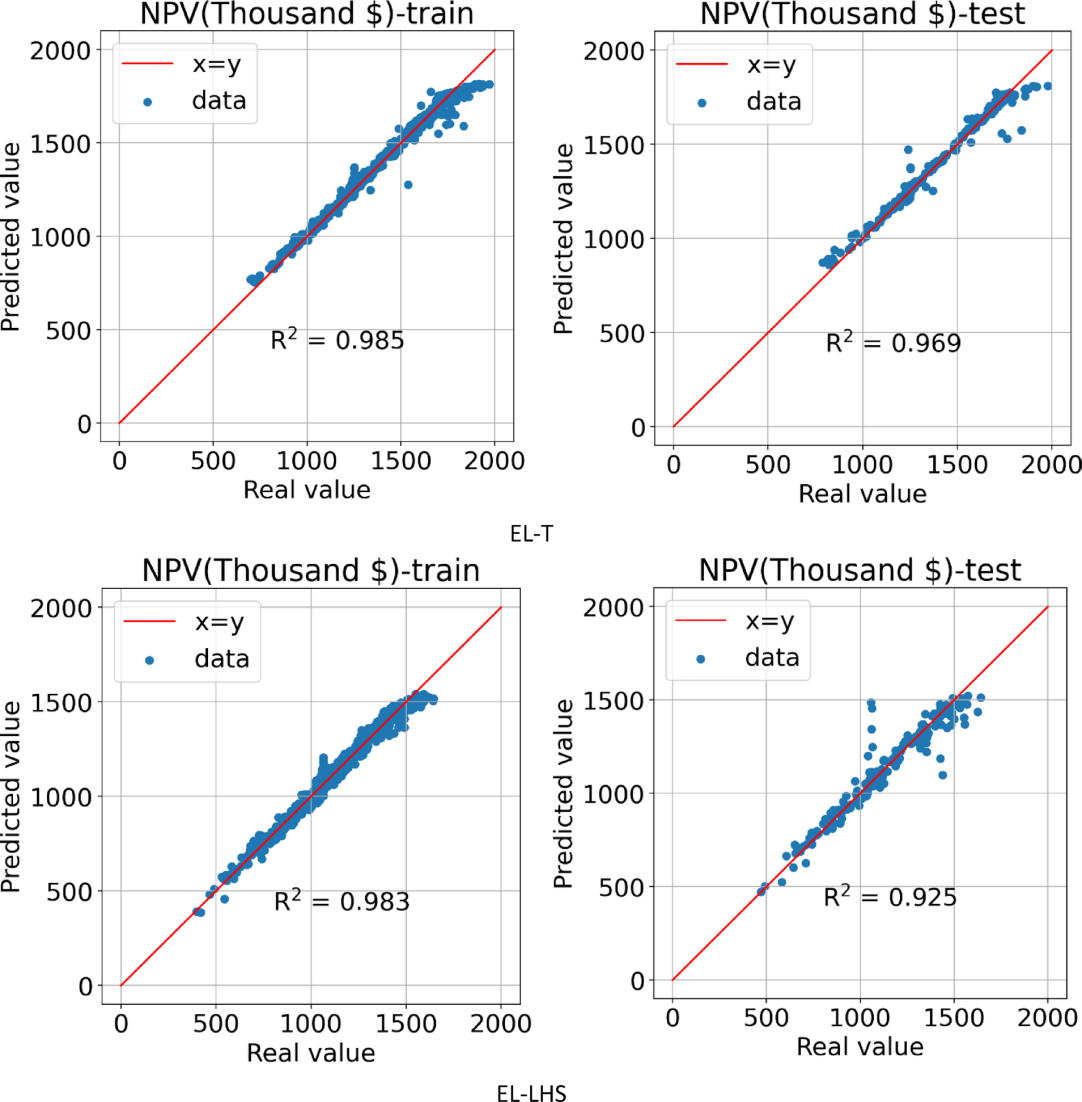
Predicted vs. real value for training and testing data obtained by EL-T & EL-LHS.

The forth method for building a proxy was to use the EL, which was performed on two datasets obtained from the LHS and Taguchi design of experiment methods. Based on the formulation provided in Eq. (2), RRMSE was calculated for each model (see Table 6). Then, the weights for each of the algorithms was determined and summarized in Table 7. After the AI ​​model learning process is complete, Fig. 17 shows the predicted NPV value in terms of the actual value for the training and testing data for the EL-LHS and EL-T proxies. In the EL-T proxy, the R^2^ values for the training, and testing data are calculated and reported as 0.985 and 0.969, respectively. For the EL-LHS proxy, the R^2^ value for the training and testing data are reported as 0.983 and 0.925, respectively, which indicates that the model accuracy was higher for the data obtained from the Taguchi design of experiment method.

**Table 6:** RRMSE for each model.

	ANN-T	ANN-LHS	LSTM-T	LSTM-LHS	GRU-T	GRU-LHS
RRMSE	0.1315	0.1732	0.0837	0.1205	0.1111	0.1066

**Table 7:** Weights for each of the algorithms.

Models	Sub models	Weights
EL-T	ANN-T	0.266
	LSTM-T	0.419
	GRU-T	0.315
EL-LHS	ANN-LHS	0.246
	LSTM-LHS	0.354
	GRU-LHS	0.400

### Models comparison

**Fig. 18:**
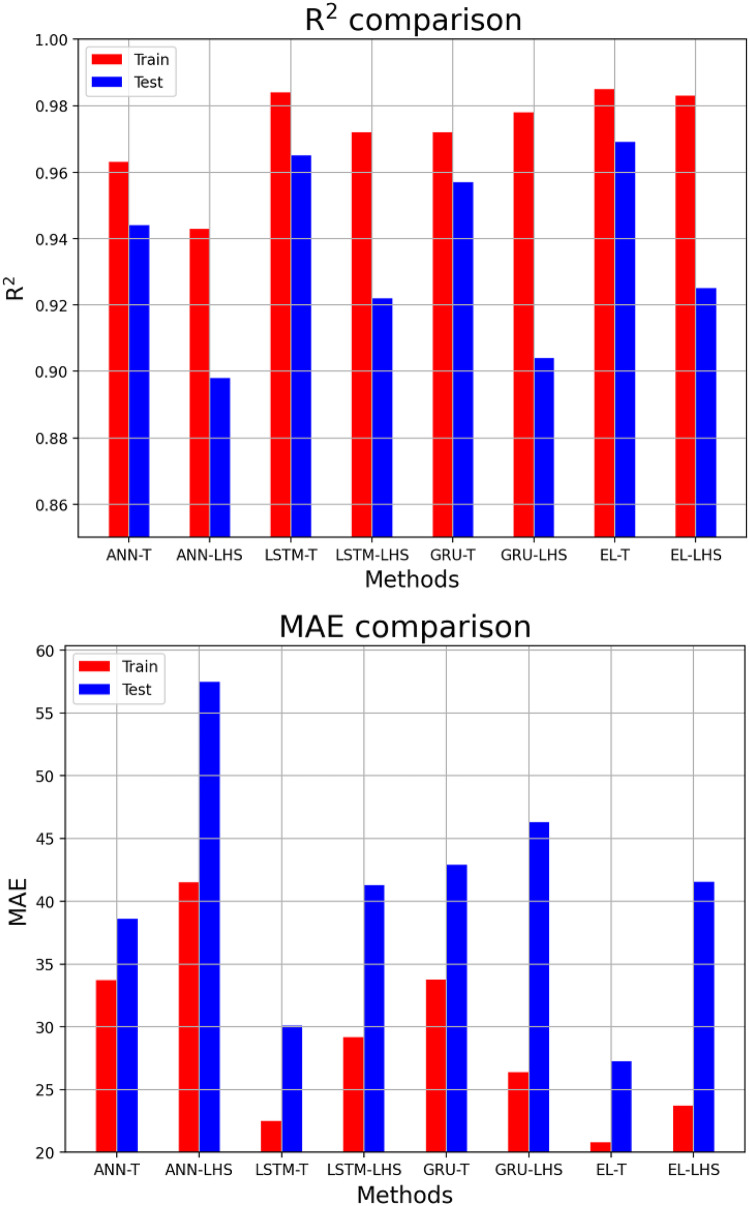
R^2^and MAE accuracies comparison for training and testing data.

**Fig. 19:**
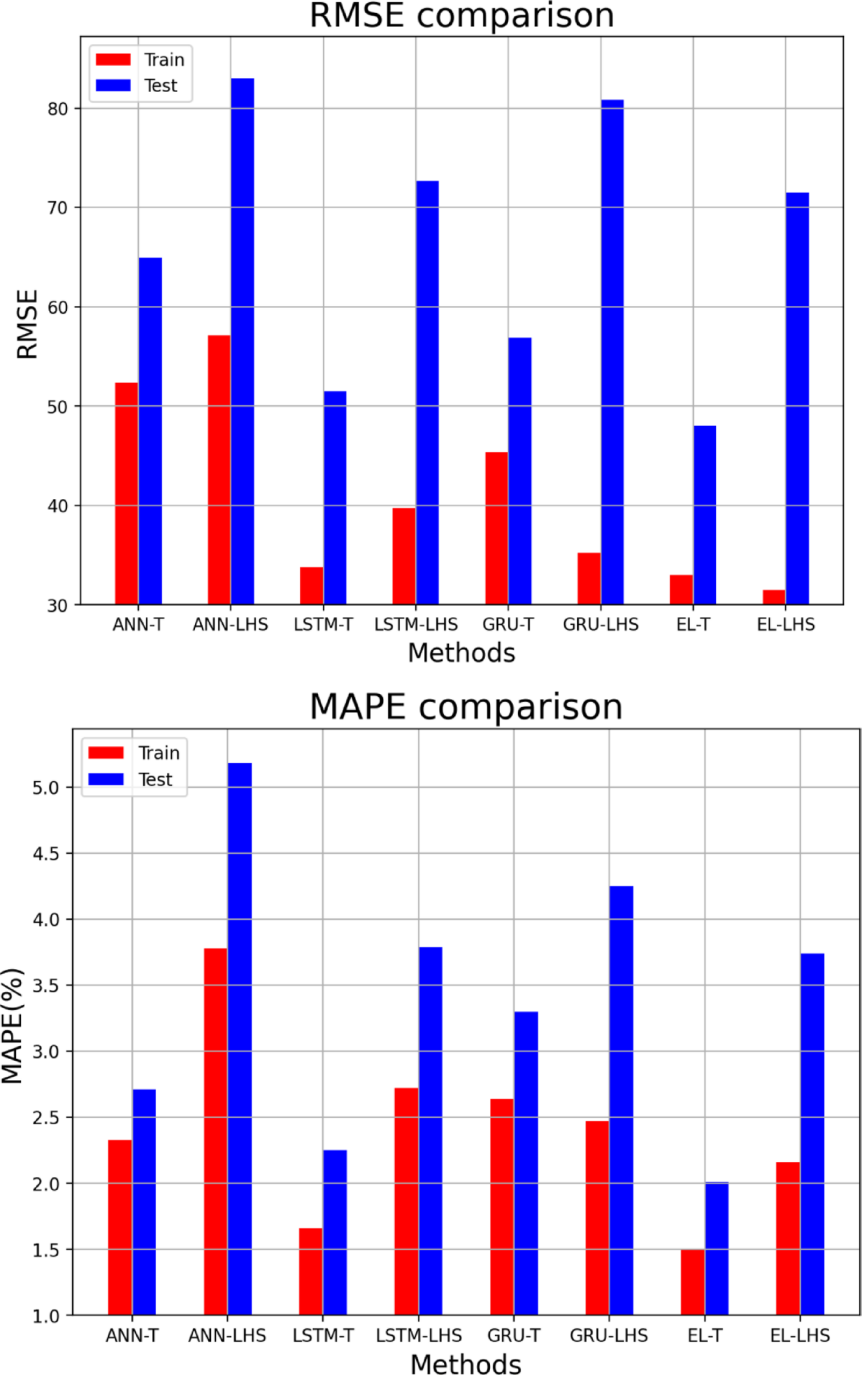
RMSE, and MAPE accuracies comparison for training and testing data.

**Fig. 20:**
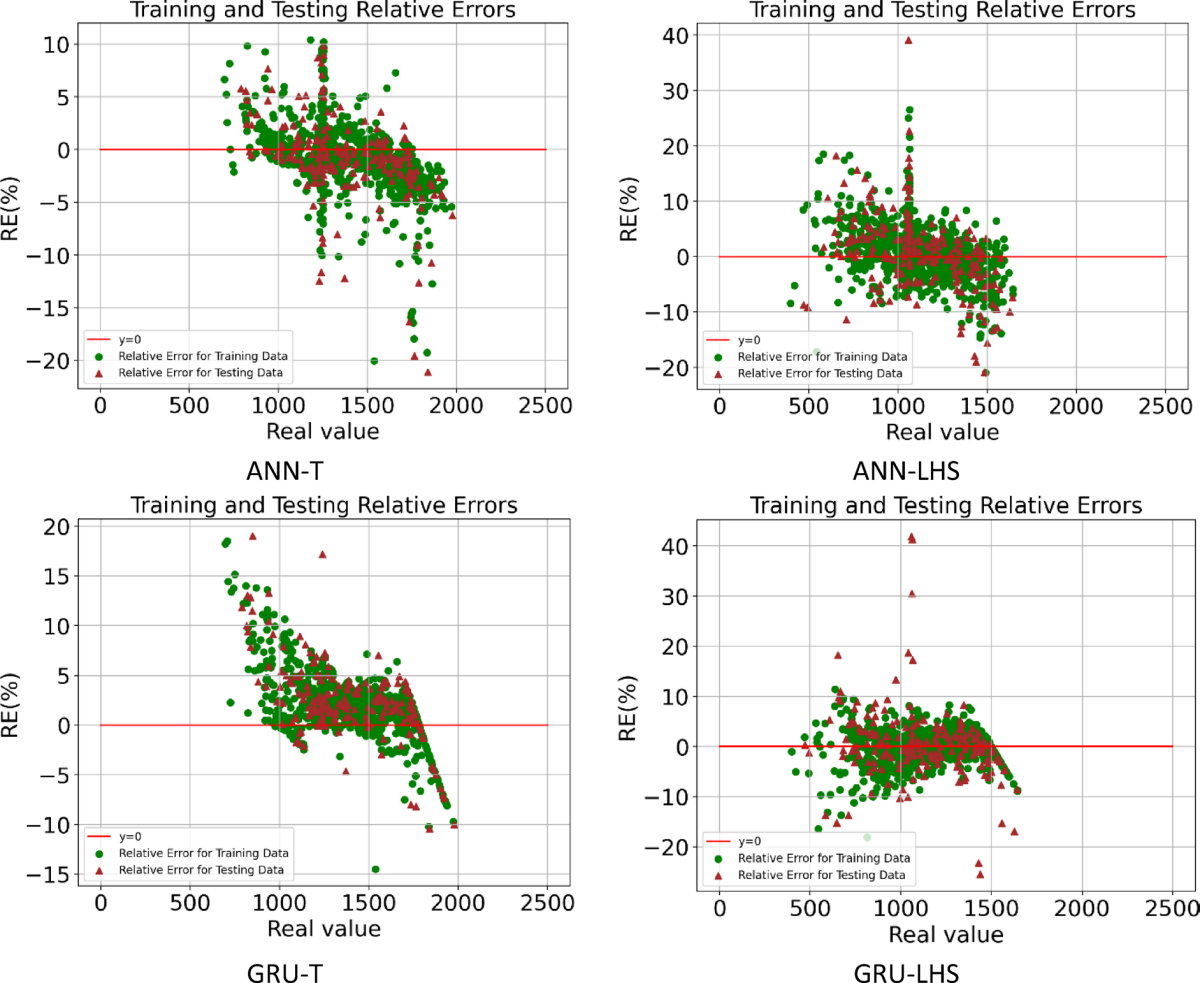
Training and testing relative errors obtained by ANN & GRU proxy models.

**Fig. 21:**
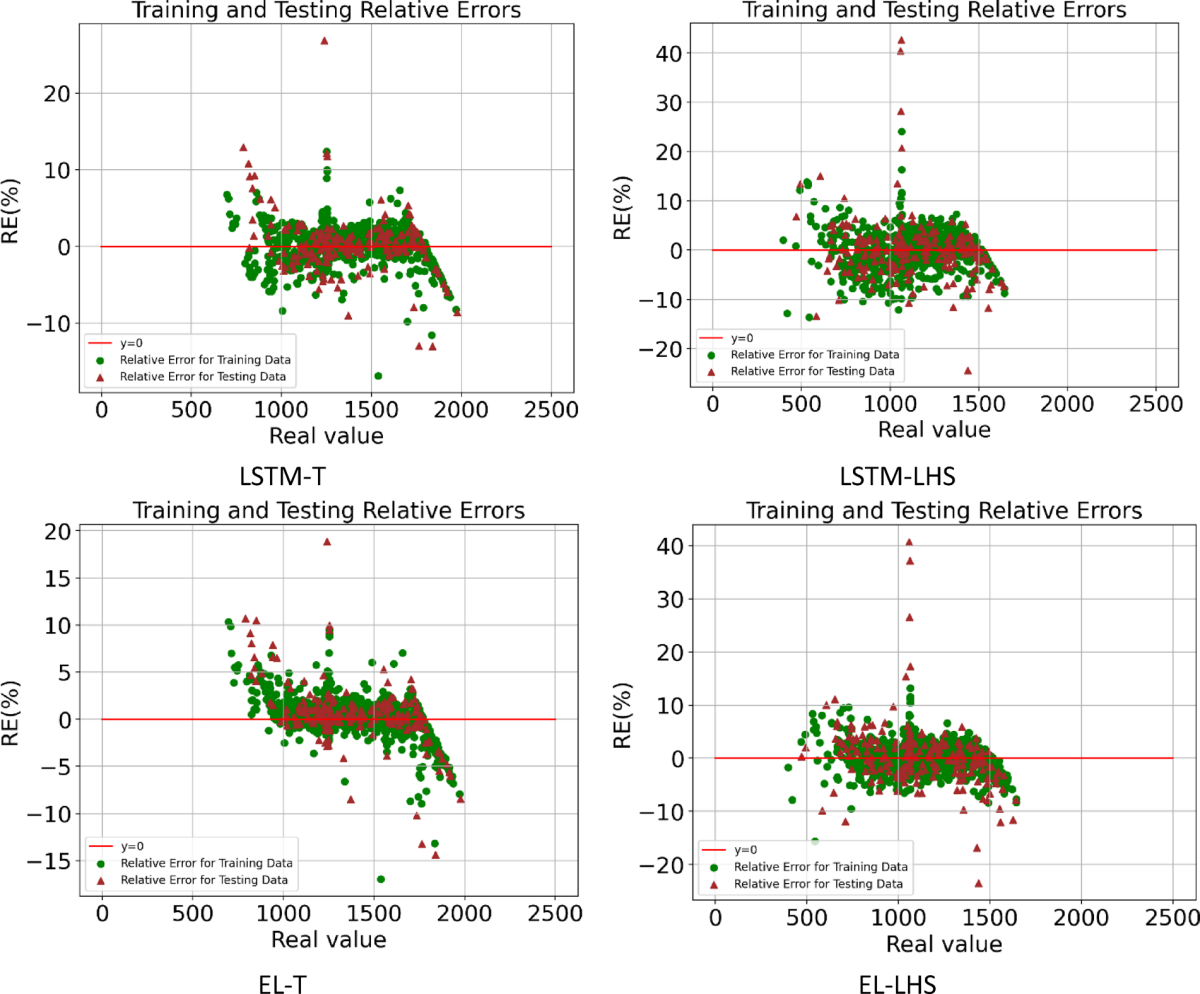
Training and testing relative errors obtained by LSTM & EL proxy models.

In this section, it is discussed the accuracy of proxy models built by four deep learning algorithms for two types of datasets. Table 8 shows the proxy accuracy of the models including R^2^MAE, RMSE, and MAPE, for the training and testing data, which can be compared schematically in Figs. 18 and 19. By comparing the obtained accuracies, it is observed that the proxy models built with the EL methods achieved the highest accuracy, and among the datasets used, the Taguchi method was able to obtain data for building a proxy with higher accuracy. Figures 20 and 21 also shows the RE for the training and testing data for eight different proxy models, which shows that the error dispersion in the data for the EL algorithm is less than the rest of the methods. If we compare all the algorithms with each other, the order is EL > LSTM > GRU > ANN, and the data set obtained from the Taguchi method has achieved greater accuracy (Taguchi > LHS).

**Table 8:** Training and testing accuracies obtained by proxy models.

Accuracy		ANN-T	ANN-LHS	LSTM-T	LSTM-LHS	GRU-T	GRU-LHS	EL-T	EL-LHS
R^2^	**Train**	0.963	0.943	0.984	0.972	0.972	0.978	0.985	0.983
	**Test**	0.944	0.898	0.965	0.922	0.957	0.904	0.969	0.925
MAE	**Train**	33.73	41.50	22.52	29.20	33.77	26.41	20.81	23.72
	**Test**	38.61	57.47	30.11	41.29	42.90	46.32	27.28	41.56
RMSE	**Train**	52.36	57.12	33.79	39.76	45.37	35.20	33.02	31.49
	**Test**	64.91	83.04	51.50	72.70	56.87	80.86	48.03	71.49
MAPE	**Train**	2.33	3.78	1.66	2.72	2.64	2.47	1.50	2.16
	**Test**	2.71	5.18	2.25	3.79	3.30	4.25	2.01	3.74

### Optimization

**Fig. 22:**
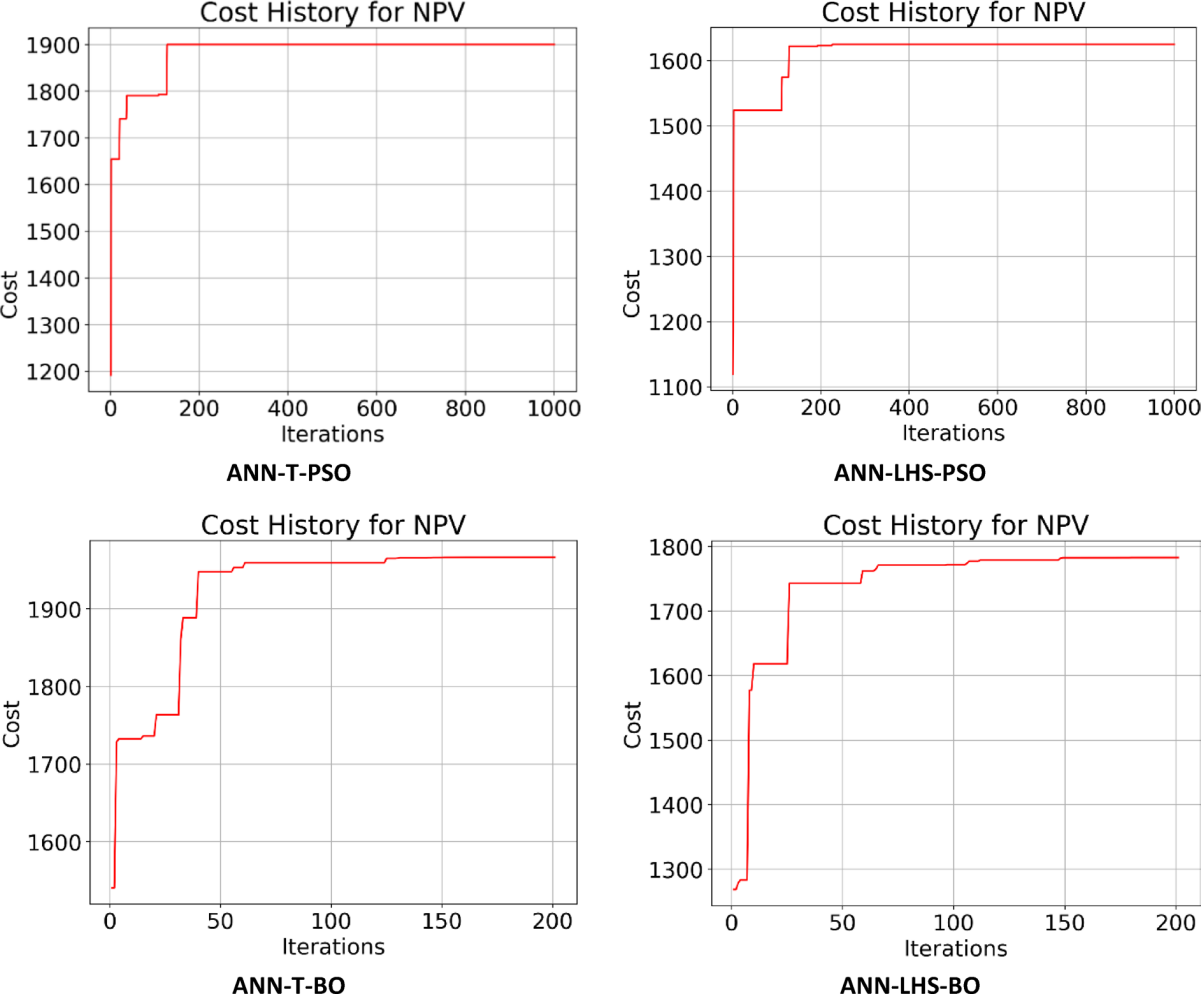
Optimization cost (Thousand $) history for NPV obtained for ANN-T & ANN-LHS.

**Fig. 23:**
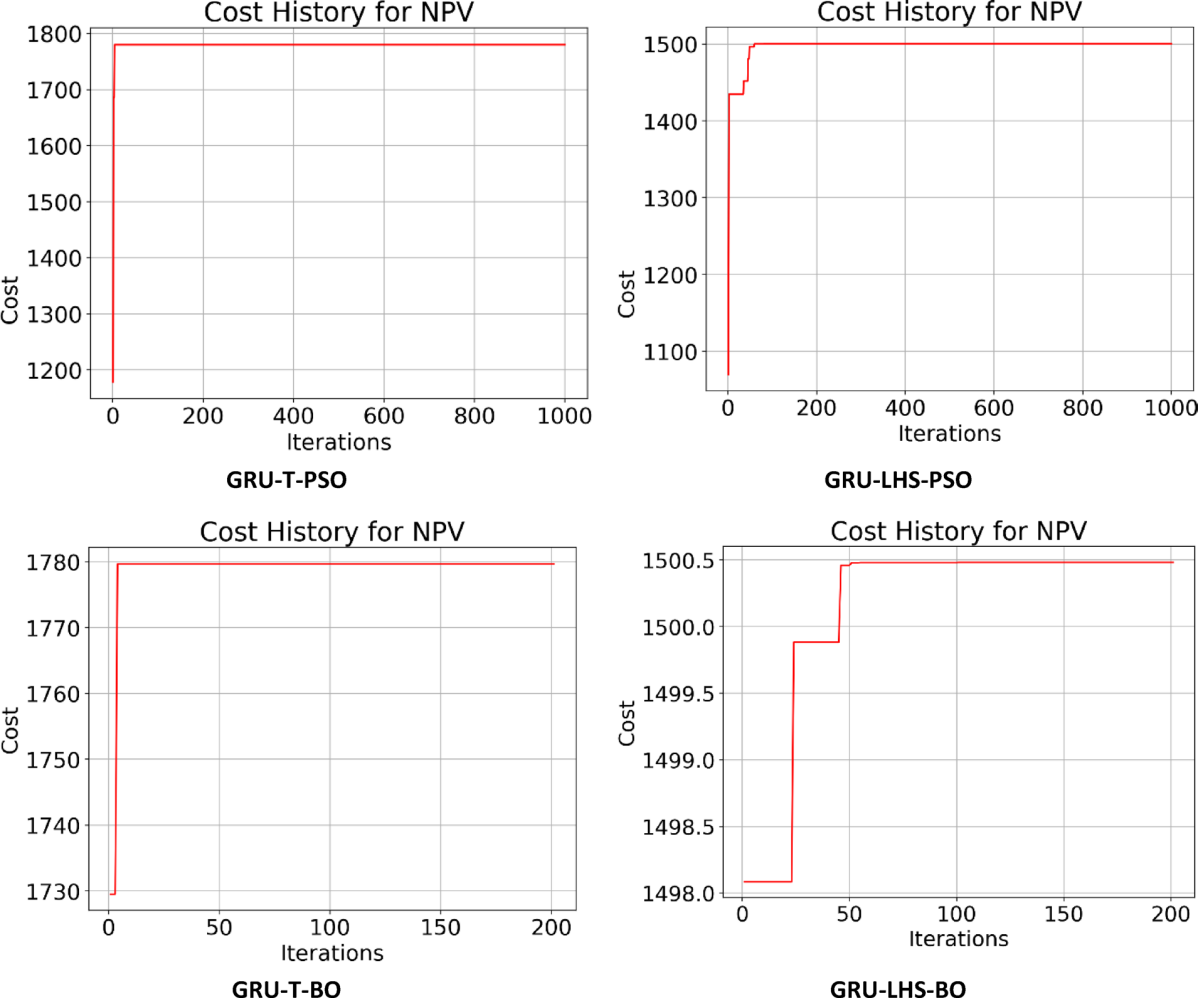
Optimization cost (Thousand $) history for NPV obtained for GRU-T & GRU-LHS.

**Fig. 24:**
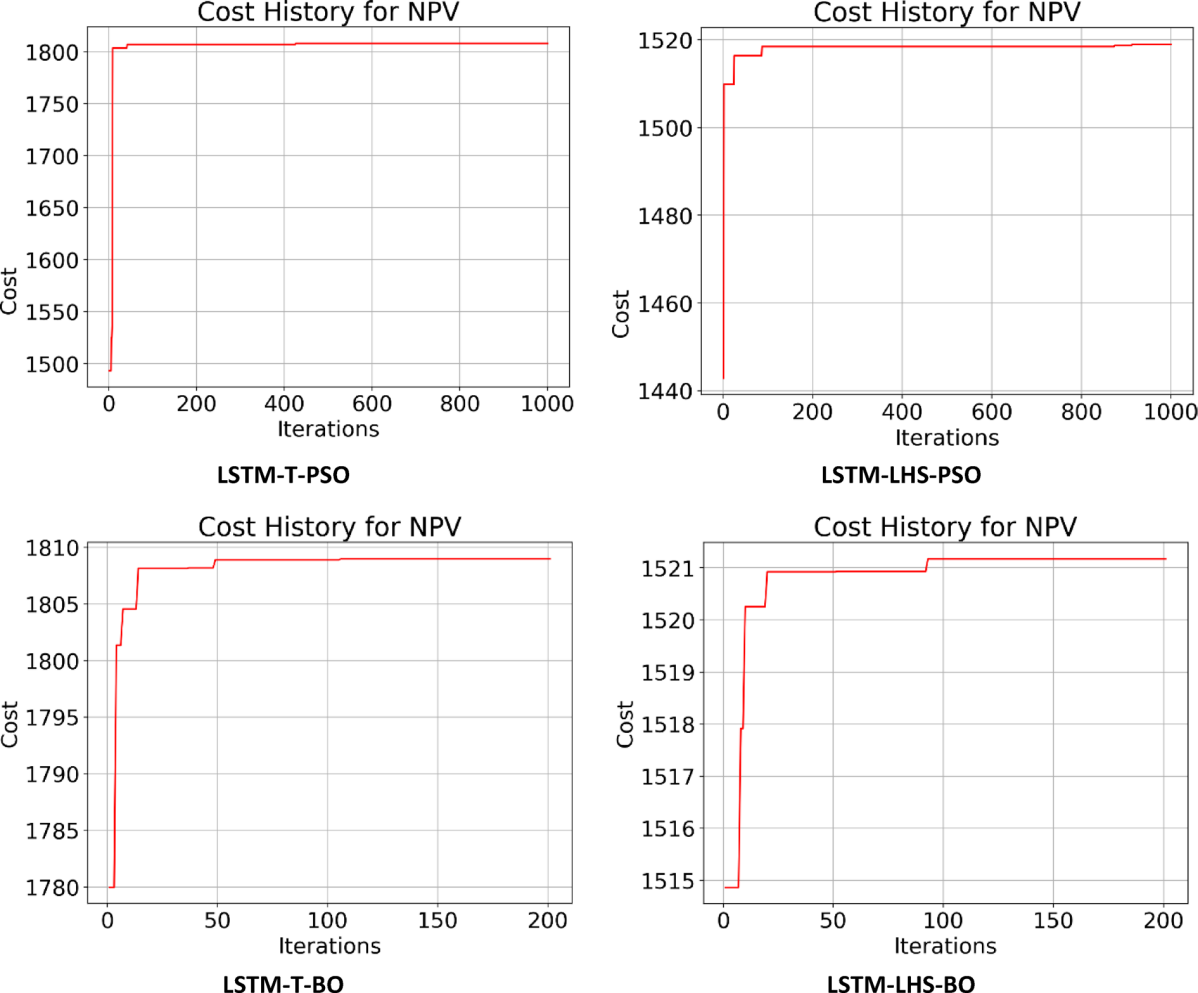
Optimization cost (Thousand $) history for NPV obtained for LSTM-T & LSTM-LHS.

**Fig. 25:**
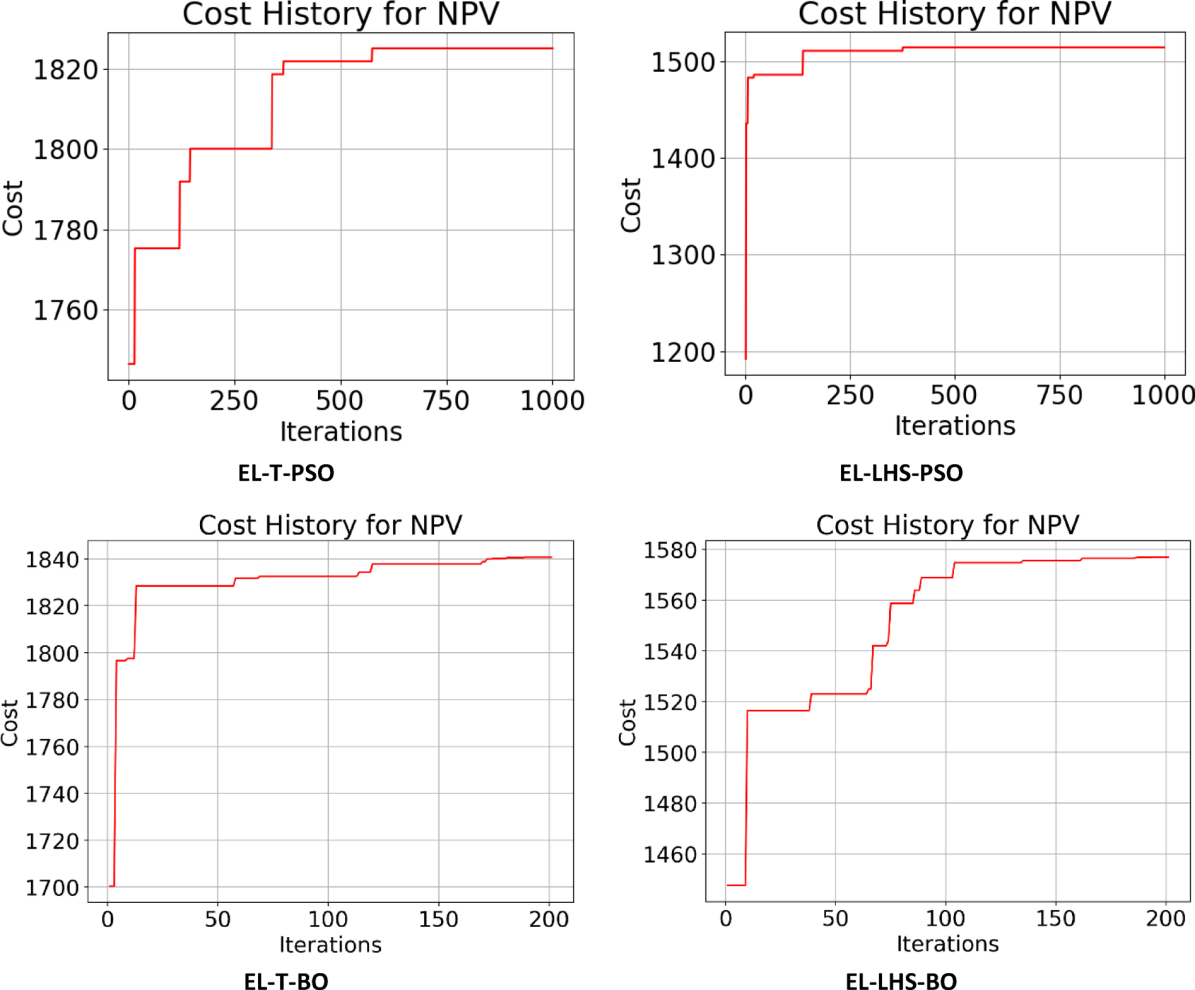
Optimization cost (Thousand $) history for NPV obtained for GRU-T & GRU-LHS.

**Fig. 26:**
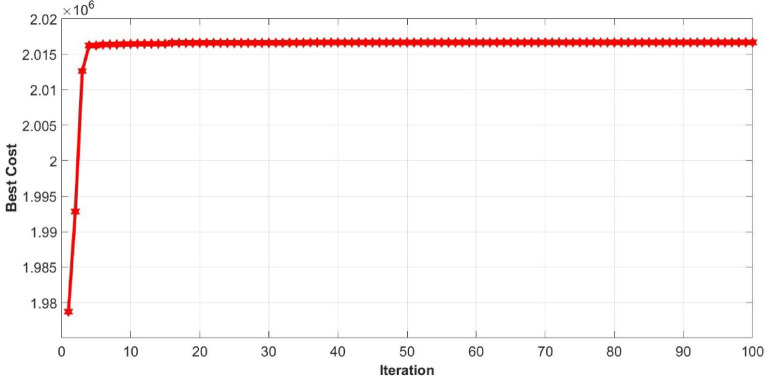
Optimization cost history for NPV from real model using PSO.

**Fig. 27:**
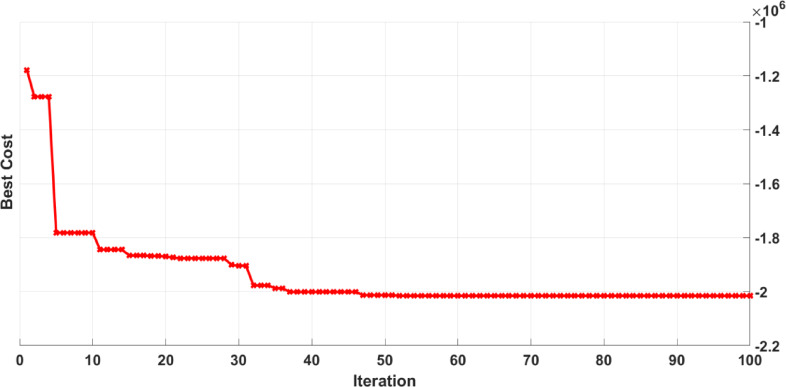
Optimization cost history for opposite of NPV from real model using BO.

Following the creation of the proxy models, they were optimized using the PSO and BO algorithms, leading to the cost history shown in Figs. 22, 23, 24 and 25. The Particle Swarm Optimization (PSO) algorithm was implemented with 1000 iterations and a swarm size of 100 particles, while the Bayesian Optimization (BO) algorithm was executed for 200 iterations. As stated, alongside the optimization with proxy models, the real reservoir model was utilized to enhance the injection and production strategy during the flooding process, as illustrated in Figs. 26 and 27, which depict the cost against iterations.

**Fig. 28:**
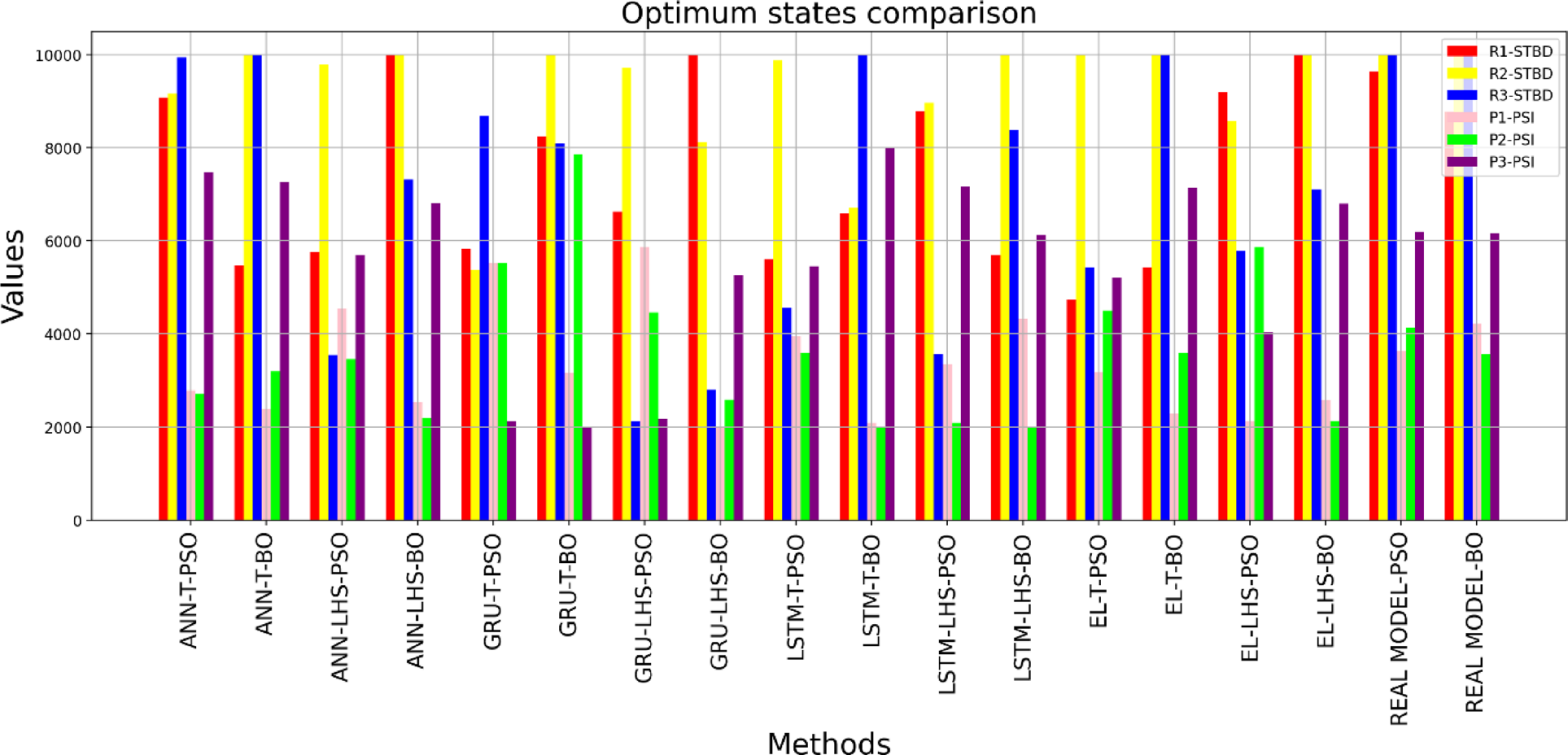
Optimum well control parameters comparison obtained by proxy models.

**Fig. 29:**
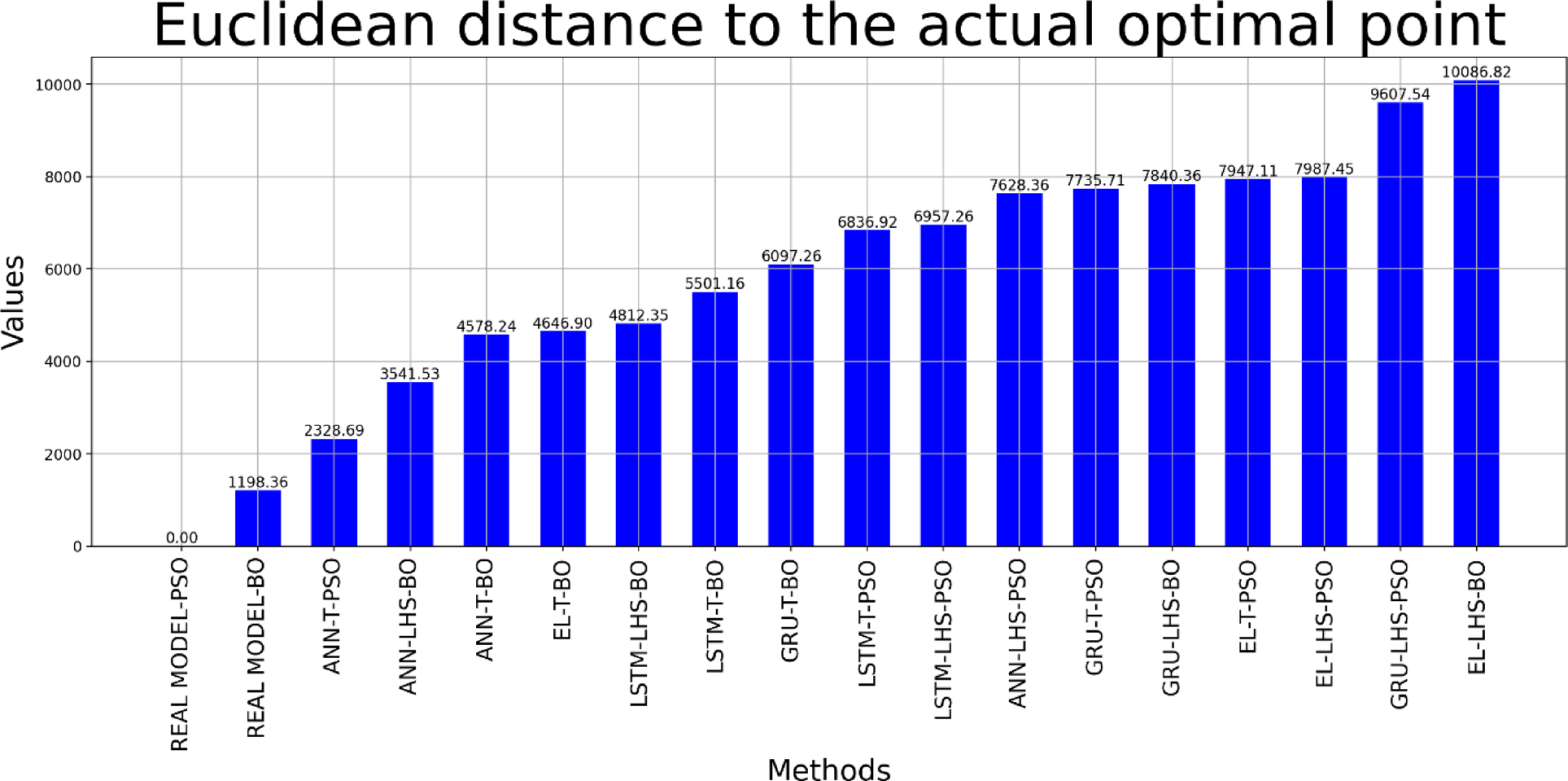
Euclidean distance of the optimal points obtained by proxy models relative to the actual optimal point.

Table 9 presents the optimization outcomes obtained through proxy models and the real model. The results obtained can be seen in Fig. 28, which indicates the variation in the optimal points in the proxy models and their difference from the optimal point obtained by the real reservoir models. Taking the optimal point obtained by the real reservoir model using PSO as the reference point, the Euclidean distance of the optimal points obtained by the eight proxy models was calculated, and this difference can be seen in Fig. 29. It is observed that the optimal point obtained by the ANN method using data obtained from the Taguchi method has the smallest Euclidean distance from the reference point among proxy models.

**Table 9:** Optimum States comparison obtained by proxy models.

Proxy	R1 (STBD)	R2 (STBD)	R3 (STBD)	P1 (psi)	P2 (psi)	P3 (psi)	Cal. NPV (Thousand $)	Actual NPV (Thousand $)
ANN-T-PSO	9072	9151	9942	2775	2713	7477	1900	1867
ANN-T-BO	5463	10,000	10,000	2388	3193	7256	1966	1898
ANN-LHS-PSO	5757	9784	3547	4547	3455	5693	1692	1890
ANN-LHS-BO	10,000	10,000	7320	2531	2205	6806	1783	1830
GRU-T-PSO	5821	5367	8683	5520	5520	2119	1768	1684
GRU-T-BO	8238	10,000	8086	3151	7845	2000	1780	1689
GRU-LHS-PSO	6626	9712	2120	5856	4452	2177	1500	1820
GRU-LHS-BO	10,000	8106	2792	2000	2583	5245	1500	1835
LSTM-T-PSO	5604	9870	4557	3935	3593	5455	1808	1889
LSTM-T-BO	6592	6712	10,000	2083	2000	8000	1809	1601
LSTM-LHS-PSO	8786	8959	3559	3338	2082	7161	1519	1778
LSTM-LHS-BO	5692	10,000	8388	4323	2000	6122	1520	1990
EL-T-PSO	4727	10,000	5414	3165	4497	5205	1811	1868
EL-T-BO	5417	10,000	10,000	2288	3578	7136	1841	1915
EL-LHS-PSO	9187	8566	5766	2134	5865	4032	1495	1784
EL-LHS-BO	10,000	10,000	7098	2580	2128	6788	1577	1949
Real model-PSO	9637	10,000	9992	3623	4128	6189	2017	2017
Real model-BO	8770	9967	9992	4214	3551	6156	2016	2016

**Fig. 30:**
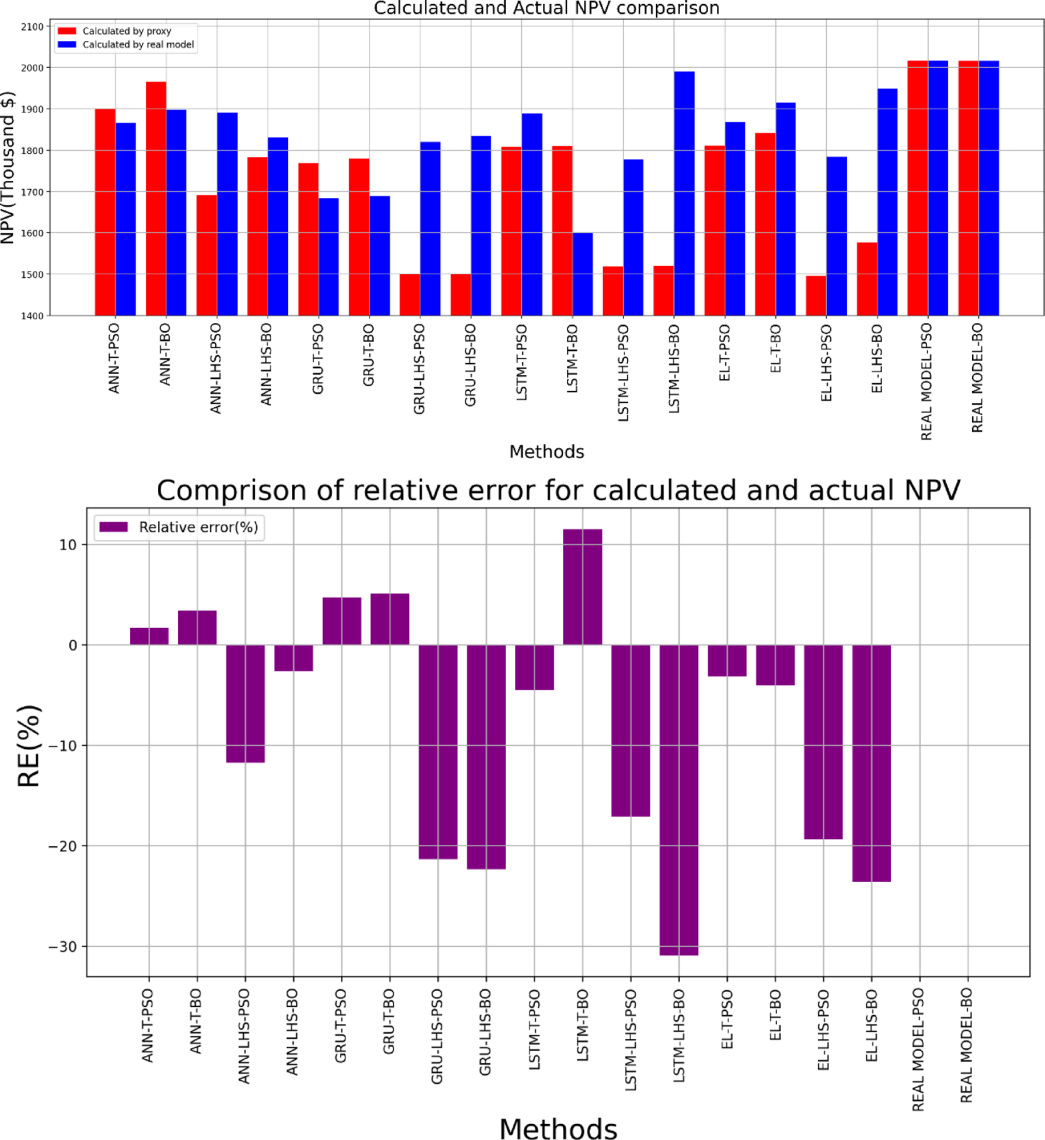
Comparison of NPV calculated by proxy models and the actual model and their relative errors for the obtained optimal conditions.

Figure 30 compares the maximum NPV values ​​obtained from proxy models and the corresponding actual NPV value with the NPV value of the reference case, and it is seen that the proxy obtained by the ANN-T proxy was able to obtain a relative lower error than the other proxies.

Discrepancies between proxy and real reservoir model results often stem from differences in data representation and model complexity. Proxy models typically rely on simplified assumptions and limited training datasets that may not fully capture the nonlinearities and heterogeneities present in real reservoirs, leading to reduced accuracy under certain conditions. Additionally, proxy models might suffer from biases introduced by uneven data distribution, especially in scenarios with rare or extreme reservoir behaviors, In contrast, real reservoir simulations incorporate detailed physics and geologic variability. Model specifics, such as the choice of surrogate algorithms and their capacity to generalize beyond training scenarios, also significantly influence performance gaps, highlighting the need for careful validation and adaptive model refinement.

## Advantages, limitations, and future work

### Advantages

This study offers a systematic evaluation of proxy model performance in the context of reservoir optimization, addressing a critical and timely challenge in reservoir engineering. By applying the workflow to the Punq-S3 benchmark model and using deep learning algorithms (ANN, LSTM, GRU and EL) combined with two design of experiment techniques (Taguchi and Latin Hypercube Sampling), the study provides a comprehensive comparison framework. The integration of particle swarm optimization (PSO) and Bayesian optimization (BO) with both proxy-based and real reservoir-based approaches allows for a meaningful evaluation of predictive reliability. Notably, the analysis identified that using the Taguchi method in combination with artificial neural networks (ANN) can yield more trustworthy proxy models, offering guidance for future model development.

### Limitations

Despite achieving high accuracy in the training and validation stages, the proxy models failed to reproduce the actual optimal control strategies obtained from the complete reservoir model. This discrepancy reveals a key limitation: the inability of some proxy models to generalize well for optimization purposes, especially in unseen scenarios. Furthermore, although proxy models reduce simulation time during the optimization stage, their training process especially for deep learning methods—can be computationally intensive and require fine-tuning. Finally, the findings are based on a single benchmark case (Punq-S3), and further validation across different reservoir types and complexities is necessary to generalize the results.

### Future work

Building on the findings of this study, future work will focus on enhancing the generalization capability and reliability of proxy models in optimization tasks. One key direction is the incorporation of hybrid modeling approaches that combine physics-based constraints with data-driven learning to improve robustness. Additionally, we plan to investigate adaptive sampling strategies to improve training data distribution and reduce bias in underrepresented regions of the input space. Expanding the study to include more complex and heterogeneous reservoir models, as well as testing the framework on real-field data, will also help validate and extend the applicability of the proposed methods. Finally, we aim to explore advanced optimization algorithms, such as reinforcement learning to better exploit the strengths of proxy models in real-time decision-making.

## Conclusions

This study examined the validity of waterflooding optimization in the Punq-s3 benchmark reservoir through proxy models and AI models. To achieve this aim, the optimization procedure was executed with eight proxy models based on deep learning algorithms (ANN, LSTM, GRU and EL) and two types of datasets(Taguchi and LHS), while concurrently, another optimization procedure utilized the real reservoir model.

Evaluating the outcomes of modeling and optimization revealed that while proxy models can predict net present value (NPV) with high precision, speed, and low computational cost, they may not fully capture complex reservoir dynamics, despite exhibiting high R² values. Therefore, while proxy models and artificial intelligence hold promise for enhancing reservoir management, their application should be carefully validated and potentially supplemented with high-fidelity simulations or field data to ensure robust and dependable decision-making.

As stated, the use of proxy models, despite having high accuracy, cannot always produce a valid model for optimization, but it is possible to build a proxy model with more reliability and near to ideal state for reservoir modeling. Considering the studies conducted in terms of the type of algorithm and the type of design of experiment method for creating dataset, it is recommended to use an ANN to build proxy models and to use the Taguchi method to create the data set for its construction. Also, in examining the accuracy, the relative error (RE) range should be reasonable and low so that the created proxy model is sufficiently valid and the result of the optimization is reasonable.

ANN-Taguchi proxy models are the most reliable and appropriate compared to other proxy models in scenarios where rapid evaluation of multiple operational strategies is required, such as early-stage field development planning or real-time production optimization. They are particularly effective when applied to reservoirs with relatively well-characterized geology and fluid properties, where the training data adequately represent the full range of operational conditions. In industry practice, these models are ideal for sensitivity analysis, uncertainty quantification, and scenario screening, especially when computational resources are limited. For future research, they are well-suited for integration with hybrid modeling frameworks that combine data-driven and physics-based approaches to improve scalability and robustness across diverse reservoir types.

## Data Availability

“All data generated or analyzed during this study are included in this published article”.

## References

[1] Islam, M. & Khan, M. *The Petroleum Engineering Handbook: Sustainable Operations* (Elsevier, 2013).

[2] Du, X. & Thakur, G. C. Lessons learned from the process of water injection management in impactful onshore and offshore carbonate reservoirs. *Energies* **17**, 3951 (2024).

[3] Aziz, A. A. A., Yusop, N. M. & Othman, N. Influence of injection rate and oil viscosity on viscous fingering–simulation prediction. *CFD Lett.* **14**, 24–42 (2022).

[4] Jia, D., Zhang, J., Li, Y., Wu, L. & Qiao, M. Recent development of smart field deployment for mature waterflood reservoirs. *Sustainability* **15**, 784 (2023).

[5] He, J. et al. Water-flooding characteristics of lithologic reservoir in Ordos basin. *Sci. Rep.* **11**, 2503 (2021). 33510305 10.1038/s41598-021-82035-4 PMC7844225

[6] Ganiyeva, A., Karabayanova, L., Pourafshary, P. & Hashmet, M. R. The performance of engineered water flooding to enhance high viscous oil recovery. *Appl. Sci.* **12**, 3893 (2022).

[7] Kazem, M. H., Hussein, M. A., Adnan, M. S., Alfarge, D. & Mansour, I. J. The performance of streamline simulation technique to mimic the waterflooding management process in oil reservoirs. *Fuel* **348**, 128556 (2023).

[8] Grema, A. S., Landa, A. C. & Cao, Y. Dynamic self-optimizing control for oil reservoir waterflooding. *IFAC-PapersOnLine* **48**, 50–55 (2015).

[9] Graczyk, K. M. & Matyka, M. Predicting porosity, permeability, and tortuosity of porous media from images by deep learning. *Sci. Rep.* **10**, 21488 (2020). 33293546 10.1038/s41598-020-78415-x PMC7722859

[10] Abdollahfard, Y., Mirabbasi, S. M., Ahmadi, M., Hemmati-Sarapardeh, A. & Ashoorian, S. Formation permeability Estimation using mud loss data by deep learning. *Sci. Rep.* **15**, 15251 (2025). 40307334 10.1038/s41598-025-94617-7 PMC12043842

[11] Lv, Q., Zheng, R., Guo, X., Larestani, A., Hadavimoghaddam, F., Riazi, M., Hemmati-Sarapardeh, A., Wang, K. and Li, J. Modelling minimum miscibility pressure of CO2-crude oil systems using deep learning, tree-based, and thermodynamic models: Application to CO2 sequestration and enhanced oil recovery. *Sep. Purif. Technol*. **310**, 123086 (2023).

[12] Ali, A. & Guo, L. Adaptive neuro-fuzzy approach for prediction of dewpoint pressure for gas condensate reservoirs. *Pet. Sci. Technol.* **38**, 673–681 (2020).

[13] Hui, G., Chen, S., He, Y., Wang, H. & Gu, F. Machine learning-based production forecast for shale gas in unconventional reservoirs via integration of geological and operational factors. *J. Nat. Gas Sci. Eng.* **94**, 104045 (2021).

[14] Liu, X., Ge, Q., Chen, X., Li, J. & Chen, Y. Extreme learning machine for multivariate reservoir characterization. *J. Petrol. Sci. Eng.* **205**, 108869 (2021).

[15] Ali, A. & Guo, L. Neuro-adaptive learning approach for predicting production performance and pressure dynamics of gas condensation reservoir. *IFAC-PapersOnLine* **52**, 122–127 (2019).

[16] Larestani, A., Mousavi, S. P., Hadavimoghaddam, F. & Hemmati-Sarapardeh, A. Predicting formation damage of oil fields due to mineral scaling during water-flooding operations: gradient boosting decision tree and cascade-forward back-propagation network. *J. Petrol. Sci. Eng.* **208**, 109315 (2022).

[17] Bai, Y., Berezovsky, V. & Popov, V. in *2020 International Conference on High Performance Big Data and Intelligent Systems (HPBD&IS).* 1–6 (IEEE).

[18] Najafi, A. et al. Upscaling permeability anisotropy in digital sandstones using convolutional neural networks. *J. Nat. Gas Sci. Eng.* **96**, 104263 (2021).

[19] Telvari, S., Sayyafzadeh, M., Siavashi, J. & Sharifi, M. Prediction of two-phase flow properties for digital sandstones using 3D convolutional neural networks. *Adv. Water Resour.* **176**, 104442 (2023).

[20] Xue, L. et al. An automated data-driven pressure transient analysis of water-drive gas reservoir through the coupled machine learning and ensemble Kalman filter method. *J. Petrol. Sci. Eng.* **208**, 109492 (2022).

[21] Khazali, N. & Sharifi, M. New approach for interpreting pressure and flow rate data from permanent downhole gauges, least square support vector machine approach. *J. Petrol. Sci. Eng.* **180**, 62–77 (2019).

[22] Wu, P. Y., Jain, V., Kulkarni, M. S. & Abubakar, A. in *2018 SEG International Exposition and Annual Meeting.* (OnePetro).

[23] Amiri-Ramsheh, B., Zabihi, R. and Hemmati-Sarapardeh, A.. Modeling wax deposition of crude oils using cascade forward and generalized regression neural networks: Application to crude oil production. *Geoenergy Sci. Eng*. **224**, 211613 (2023).

[24] Moncayo-Riascos, I. et al. Integrated machine learning model for predicting asphaltene damage risk and the asphaltene onset pressure. *Energy Fuels*. **36**, 14243–14252 (2022).

[25] Khodabakhshi, M. J. & Bijani, M. Predicting scale deposition in oil reservoirs using machine learning optimization algorithms. *Results Eng.* **22**, 102263 (2024).

[26] Khamehchi, E., Kivi, I. R. & Akbari, M. A novel approach to sand production prediction using artificial intelligence. *J. Petrol. Sci. Eng.* **123**, 147–154 (2014).

[27] Ali, A., Aliyuda, K., Elmitwally, N. & Bello, A. M. Towards more accurate and explainable supervised learning-based prediction of deliverability for underground natural gas storage. *Appl. Energy*. **327**, 120098 (2022).

[28] Yousefzadeh, R. & Ahmadi, M. Improved history matching of channelized reservoirs using a novel deep learning-based parametrization method. *Geoenergy Sci. Eng.* **229**, 212113 (2023).

[29] Yousefzadeh, R. & Ahmadi, M. Fast marching method assisted permeability upscaling using a hybrid deep learning method coupled with particle swarm optimization. *Geoenergy Sci. Eng.* **230**, 212211 (2023).

[30] Alfarizi, M. G., Stanko, M. & Bikmukhametov, T. Well control optimization in waterflooding using genetic algorithm coupled with artificial neural networks. *Upstream Oil Gas Technol.* **9**, 100071 (2022).

[31] Zhang, K. et al. A double-model differential evolution for constrained waterflooding production optimization. *J. Petrol. Sci. Eng.* **207**, 109059 (2021).

[32] Farahi, M. M., Ahmadi, M. & Dabir, B. Model-based water-flooding optimization using multi-objective approach for efficient reservoir management. *J. Petrol. Sci. Eng.* **196**, 107988 (2021).

[33] Ng, C. S. W. & Jahanbani Ghahfarokhi, A. Nait amar, M. Application of nature-inspired algorithms and artificial neural network in waterflooding well control optimization. *J. Petroleum Explor. Prod. Technol.* **11**, 3103–3127 (2021).

[34] Ng, C. & Jahanbani Ghahfarokhi, A. & Nait Amar, M. (2022).

[35] Ng, C. S. W., Ghahfarokhi, A. J. & Amar, M. N. Production optimization under waterflooding with long Short-Term memory and metaheuristic algorithm. *Petroleum* **9**, 53–60 (2023).

[36] Ng, C. S. W., Amar, M. N., Ghahfarokhi, A. J. & Imsland, L. A survey on the application of machine learning and metaheuristic algorithms for intelligent proxy modeling in reservoir simulation. *Comput. Chem. Eng.* **170**, 108107 (2023).

[37] Matthew, D. A. M., Ghahfarokhi, J. & Ng, A. Nait amar, M. Proxy model development for the optimization of water alternating CO2 gas for enhanced oil recovery. *Energies* **16**, 3337 (2023).

[38] Jaber, A. K., Alhuraishawy, A. K. & AL-Bazzaz, W. H. in *SPE Kuwait Oil and Gas Show and Conference.* D033S013R002 (SPE).

[39] Brouwer, D. R. & Jansen, J. D. Dynamic optimization of waterflooding with smart wells using optimal control theory. *SPE J.* **9**, 391–402 (2004).

[40] Toutenburg, H., Shalabh, H. & Shalabh, H. Statistical analysis of designed experiments. (2002).

[41] Taguchi, G. *Introduction to quality engineering: designing quality into products and processes*. (1986).

[42] Roy, R. K. *A Primer on the Taguchi Method* (Society of manufacturing engineers, 2010).

[43] Dean, A. & Voss, D. *Design and Analysis of Experiments* (Springer, 1999).

[44] McKay, M. D., Beckman, R. J. & Conover, W. J. A comparison of three methods for selecting values of input variables in the analysis of output from a computer code. *Technometrics* **42**, 55–61 (2000).

[45] Santner, T. J., Williams, B. J., Notz, W. I. & Williams, B. J. *The Design and Analysis of Computer Experiments*Vol. 1 (Springer, 2003).

[46] Helton, J. C. & Davis, F. J. Latin hypercube sampling and the propagation of uncertainty in analyses of complex systems. *Reliab. Eng. Syst. Saf.* **81**, 23–69 (2003).

[47] Tarantola, S., Gatelli, D. & Mara, T. A. Random balance designs for the Estimation of first order global sensitivity indices. *Reliab. Eng. Syst. Saf.* **91**, 717–727 (2006).

[48] Forrester, A., Sobester, A. & Keane, A. *Engineering Design Via Surrogate Modelling: a Practical Guide* (Wiley, 2008).

[49] Hassoun, M. H. *Fundamentals of Artificial Neural Networks* (MIT Press, 1995).

[50] LeCun, Y., Bengio, Y. & Hinton, G. Deep learning. *Nature* **521**, 436–444 (2015). 26017442 10.1038/nature14539

[51] Bishop, C. M. *Neural Networks for Pattern Recognition* (Oxford University Press, 1995).

[52] Rumelhart, D. E., Hinton, G. E. & Williams, R. J. Learning representations by back-propagating errors. *nature* 323, 533–536 (1986).

[53] Schmidhuber, J. Deep learning in neural networks: an overview. *Neural Netw.* **61**, 85–117 (2015). 25462637 10.1016/j.neunet.2014.09.003

[54] Van Houdt, G., Mosquera, C. & Nápoles, G. A review on the long short-term memory model. *Artif. Intell. Rev.* **53**, 5929–5955 (2020).

[55] Li, Y., Sun, R., Horne, R. & R. in *SPE Annual Technical Conference and Exhibition?* D011S008002 (SPE).

[56] Chung, J., Gulcehre, C., Cho, K. & Bengio, Y. Empirical evaluation of gated recurrent neural networks on sequence modeling. *arXiv preprint arXiv:1412.3555* (2014).

[57] Zhou, Z. H. *Ensemble Methods: Foundations and Algorithms* (CRC, 2025).

[58] Dietterich, T. G. in *International workshop on multiple classifier systems.* 1–15 (Springer).

[59] Chen, S. & Luc, N. M. RRMSE Voting Regressor: A weighting function based improvement to ensemble regression. *arXiv preprint arXiv:2207.04837* (2022).

[60] Kennedy, J. & Eberhart, R. in *Proceedings of ICNN’95-international conference on neural networks.* 1942–1948 (ieee).

[61] Shahriari, B., Swersky, K., Wang, Z., Adams, R. P. & De Freitas, N. Taking the human out of the loop: A review of Bayesian optimization. *Proceedings of the IEEE* 104, 148–175 (2015).

[62] Kennedy, J. Particle swarm optimization. Encyclopedia of machine learning. *Springer* **760**, 766 (2011).

[63] Engelbrecht, A.P. Computational intelligence: an introduction (Vol. 2). (Hoboken, NJ, USA: John Wiley & Sons, 2007).

[64] Thangaraj, R., Pant, M., Abraham, A. & Bouvry, P. Particle swarm optimization: hybridization perspectives and experimental illustrations. *Appl. Math. Comput.* **217**, 5208–5226 (2011).

[65] Mahmoodabadi, M. J., Safaie, A. A. & Bagheri, A. Nariman-Zadeh, N. A novel combination of particle swarm optimization and genetic algorithm for Pareto optimal design of a five-degree of freedom vehicle vibration model. *Appl. Soft Comput.* **13**, 2577–2591 (2013).

[66] Clerc, M. & Kennedy, J. The particle swarm-explosion, stability, and convergence in a multidimensional complex space. *IEEE Trans. Evol. Comput.* **6**, 58–73 (2002).

[67] Snoek, J., Larochelle, H. & Adams, R. P. Practical bayesian optimization of machine learning algorithms. *Advances Neural Inform. Process. Systems Vol.* **25** (2012).

[68] Mockus, J. The application of bayesian methods for seeking the extremum. *Towards Global Optim.* **2**, 117 (1998).

[69] Jones, D. R. A taxonomy of global optimization methods based on response surfaces. *J. Global Optim.* **21**, 345–383 (2001).

[70] Rasmussen, C. E. *In Summer School on Machine Learning*63–71 (Springer, 2003).

[71] Kushner, H. J. A New Method of Locating the Maximum Point of an Arbitrary Multipeak Curve in the Presence of Noise. *ASME. J. Basic Eng.*. **86**(1), 97–106 (1964).

[72] Mockus, J. in *System Modeling and Optimization: Proceedings of the 10th IFIP Conference New York City, USA, August 31–September 4*,. 473–481 (Springer). 473–481 (Springer). (1981).

[73] Srinivas, N., Krause, A., Kakade, S. M. & Seeger, M. Gaussian process optimization in the bandit setting: No regret and experimental design. *arXiv preprint arXiv:0912.3995* (2009).

[74] Kuo, J. T., Hsieh, M. H., Lung, W. S. & She, N. Using artificial neural network for reservoir eutrophication prediction. *Ecol. Model.* **200**, 171–177 (2007).

[75] Willmott, C. J. Some comments on the evaluation of model performance. *Bull. Am. Meteorol. Soc.* **63**, 1309–1313 (1982).

[76] McKenzie, J. Mean absolute percentage error and bias in economic forecasting. *Econ. Lett.* **113**, 259–262 (2011).

[77] De Myttenaere, A., Golden, B., Le Grand, B. & Rossi, F. Mean absolute percentage error for regression models. *Neurocomputing* **192**, 38–48 (2016).

[78] Li, X. R. & Zhao, Z. in *7th international conference on information fusion.* 8 pp. (IEEE). 8 pp. (IEEE). (2005).

